# Late Repression of NF-κB Activity by Invasive but Not Non-Invasive Meningococcal Isolates Is Required to Display Apoptosis of Epithelial Cells

**DOI:** 10.1371/journal.ppat.1002403

**Published:** 2011-12-01

**Authors:** Ala-Eddine Deghmane, Hela El Kafsi, Dario Giorgini, Aziza Abaza, Muhamed-Kheir Taha

**Affiliations:** Institut Pasteur, Invasive Bacterial Infections Unit, Paris, France; Yale University School of Medicine, United States of America

## Abstract

Meningococcal invasive isolates of the ST-11 clonal complex are most frequently associated with disease and rarely found in carriers. Unlike carriage isolates, invasive isolates induce apoptosis in epithelial cells through the TNF-α signaling pathway. While invasive and non-invasive isolates are both able to trigger the TLR4/MyD88 pathway in lipooligosaccharide (LOS)-dependant manner, we show that only non-invasive isolates were able to induce sustained NF-κB activity in infected epithelial cells. ST-11 invasive isolates initially triggered a strong NF-κB activity in infected epithelial cells that was abolished after 9h of infection and was associated with sustained activation of JNK, increased levels of membrane TNFR1, and induction of apoptosis. In contrast, infection with carriage isolates lead to prolonged activation of NF-κB that was associated with a transient activation of JNK increased TACE/ADAM17-mediated shedding of TNFR1 and protection against apoptosis. Our data provide insights to understand the meningococcal duality between invasiveness and asymptomatic carriage.

## Author Summary

Strains of Neisseria meningitidis isolated from patients induce apoptotic cell death through the TNF-α pathway, whereas strains isolated from healthy carriage isolates do not. Part of the difference has been shown to arise from differential shedding of the type 1 TNF-α receptor (TNFR1) from the surface of the cells infected with the carriage isolates. Here, we elucidate some of the downstream signaling that differs between these isolates, specifically showing that carriage isolates induce sustained NF-κB activity, leading to cytoprotective events whereas invasive isolates block this NF-κB activity and thus fail to induce the downstream protective events.

## Introduction

The exclusive human bacterium *Neisseria meningitidis* (the meningococcus) is a major cause of infectious diseases worldwide, including meningitis and fulminant sepsis that are associated with significant morbidity and case fatality rates ranging from 10 to 50% in patients with severe septicaemia [1], [2] and up to 20% of survivors sustain neurological sequelae [3]. Despite this notoriety, *N. meningitidis* is a frequent inhabitant of the nasopharyngeal mucosa being asymptomatically carried by 10–35% of the adult population [4], [5]. A combination of bacterial factors and host susceptibility (including age, prior viral infection, and genetic polymorphisms [6]–[8]), may ultimately lead to meningococcal disease. Multilocus sequence typing (MLST) has been used to characterize the genotypes of meningococcal isolates determined by the sequence types (STs) and grouping these genotypes into distinct phylogenetic lineages referred to as “clonal complexes” [9]. Comparisons of the genotypes of meningococcal isolates have shown that asymptomatic carriage isolates are genetically and antigenically highly diverse, whereas most meningococcal disease is caused by a limited number of clonal complexes known as the “hyper-invasive clonal complexes” [10]–[13]. Genomic analysis failed to identify which bacterial features are responsible for the different epidemiologies [14]. Moreover, bacterial virulence factors such as pili and capsule, although important in the establishment of the disease, are widely distributed among carriage and invasive isolates. Therefore, a better understanding of the pathogenesis of this disease, notably the interaction with host cells, is central in developing new anti-meningococcal strategies.

There is increasing evidence that invasive meningococcal infections lead to cytopathic effects [15]–[20]. These observations are consistent with the extensive cell injury and tissue damage seen in autopsy material from cases of human disease [21]. We have recently shown a strong association of cytopathic effect to epithelial cells with isolates belonging to the hyper-invasive clonal complex ST-11. Infected cells presented features of apoptosis. The apoptotic pathway induced by these isolates is mediated in part by lipooligosaccharides (LOS), the major bacterial endotoxin, and involved tumor necrosis factor alpha (TNF-α) signaling through its cognate receptor TNFR1. In contrast, carriage isolates interfered with TNF-α-dependent apoptotic signaling by increasing extracellular shedding of TNFR1 leading to attenuation of the biological activity of TNF-α [22]. Several signaling pathways are known to regulate apoptosis, but the transcription factor NF-κB lies at the nexus of both anti-apoptotic and proinflammatory cascades (reviewed in references [23], [24]). In resting cells, NF-κB is sequestered in the cytosol through interactions with its inhibitor, IκB. Proinflammatory stimuli, such as lipopolysaccharide (LPS) and TNF-α, activate a signaling pathway that results in phosphorylation and subsequent degradation of IκB by the proteasome machinery. The liberated NF-κB translocates then to the nuclear compartment, where it activates the transcription of both proinflammatory and anti-apoptotic genes [25]. Given the role of NF-κB in both inflammation and apoptosis, it is not surprising that certain pathogens have also evolved mechanisms to modulate NF-κB activity during infection [26]. In this work we aimed to explore the differential ability of meningococcal invasive and non-invasive isolates to modulate the NF-κB activity.

## Results

### TLR4 contribute to apoptosis induced by the invasive ST-11 isolates

**Figure 1:**
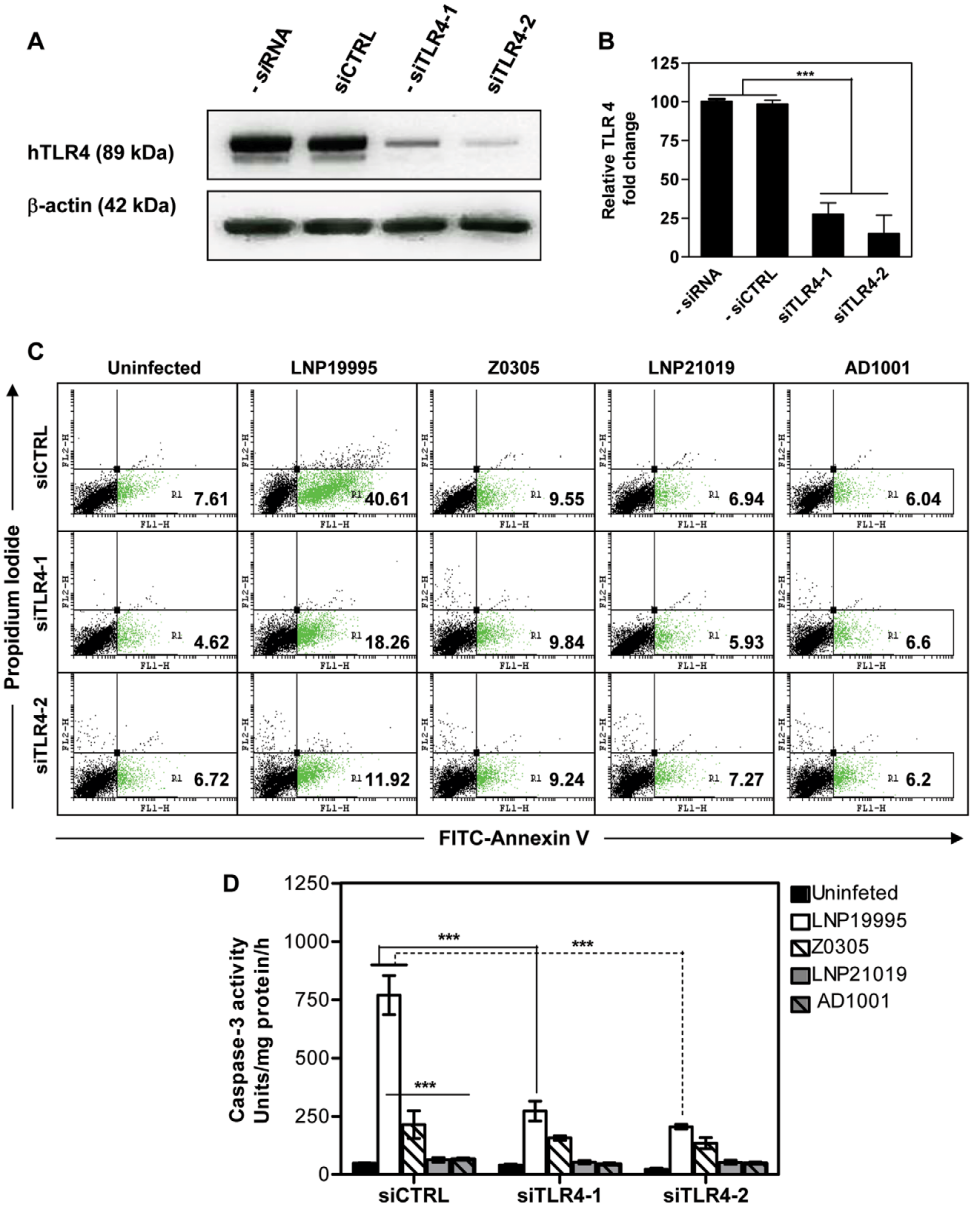
TLR4 depletion improves survival of cells infected with the ST-11 isolates. (A) Representative immunoblot of whole cell lysates from cells transfected with control (siCTRL) or TLR4 siRNAs (siTLR4-1 or siTLR4-2) or untransfected (-siRNA) blotted for TLR4 and β-actin analysis. (B) Quantification of the immunoblot in (A) showing a reduction of TLR4 expression in siTLR4-transfected but not siCTRL-transfected nor untransfected cells. Transfected cells were either infected with the indicated strains or left uninfected. After 9 h of incubation, apoptosis was assessed by PI and FITC-labeled annexin V staining and flow cytometry (C) and caspase-3 activity (D). The percentage of apoptotic cells are presented as inserts in the quadrant region R1. The data shown are representative of three independent experiments. ***, *P*<0.001.

We have shown that apoptosis of epithelial cells promoted by the ST-11 isolates is partially dependent on the meningococcal lipooligosaccharide LOS [22]. Indeed, both invasive ST-11 isolates and the non-invasive carriage isolates were able to induce the expression of TLR4 at the surface of Hec-1B epithelial cells (Supporting Figure S1). However, only invasive ST-11 isolates but not non-invasive carriage isolates or LOS-devoid mutants were able to induce apoptosis in Hec-1B epithelial cells (Figure 1C and Table 1). Anti-TLR4 neutralizing mAb but not an isotype-matching IgG control Ab was able to inhibit apoptosis in epithelial cells infected by the ST-11 invasive isolates (Table 1). Furthermore, the induction of apoptosis was abolished when TLR4 was knocked-down by *si*RNA-mediated TLR4 silencing strategy using specific TLR4 silencing duplex oligonucleotides *si*TLR4-1 or *si*TLR4-2 but not a non-specific control oligonucleotide (*si*CTRL) (Supporting Table S1 and Figures 1A and 1B). The induction of apoptosis by the invasive isolate LNP19995 was correlated with a significant high level of caspases-3 activity that was significantly reduced in the isogenic LOS-devoid mutant Z0305 or upon *si*RNA-mediated TLR4 silencing (Figure 1D). Caspase-3 activity in cells infected with the carriage isolate or its isogenic LOS mutant AD1001 were comparable to the background level. Altogether, these data demonstrate that TLR4 is required to promote apoptosis by the ST-11 meningococcal isolates. The following experiments were performed using LNP19995 and LNP21019 as a representative candidate of each, the invasive ST-11 isolates and the non-invasive carriage isolates respectively, unless otherwise specified.

**Table 1:** Role of TLR4 in apoptosis promoted by the ST-11 isolates.

	Isolates	% Apoptosis		
		No treatment	+ Irr mAb	+Anti-TLR4 mAb
	Uninfected	6,44±1,14	9,51±1,08	7,63±2,85
**Invasive ST-11**	LNP13143	36.12±1.60	37.43±2.92	15.18±2.21
	LNP17592	47.43±2.92	45.43±3.09	15.74±1.94
	LNP19008	52.18±8.22	43.62±4.24	13.24±1.24
	LNP19995	51.37±5.66	40.37±3.89	17.31±3.10
	LNP20342	42.43±0.80	42.56±5.22	18.49±3.00
	LNP20553	41.70±19.18	46.93±1.51	16,24±3,36
	LNP21515	45.18±1.15	43.50±4.24	11.31±4.16
	LNP21678	27.06±3.27	21.31±6.63	8.18±2.03
	LNP21996	39.56±1.50	31.75±8.84	22.19±11.22
	LNP24198	51.12±6.01	45.18±28.02	20.37±8.31
**Carriage**	1026	11.25±2.12	9.31±1.14	6.18±1.32
	1046	11.81±0.08	7.81±2.91	9.10±4.10
	1288	8.37±2.47	8.12±0.35	7.35±1.41
	1934	8.12±2.83	9.37±0.18	10.33±0.47
	3503	7.70±0.44	10.37±0.35	8.89±1.00
	LNP10820	10.24±2.30	7.31±0.08	3.56±1.50
	LNP16239	8.31±0.44	5.87±0.35	3.37±1.41
	LNP18166	7.93±0.09	8.99±0.88	4.19±0.62
	LNP20642	10.50±2.30	7.06±0.80	7.12±7.60
	LNP21019	7.50±3.71	9.25±2.12	6.67±1.87

Hec-B cells were treated with anti-TLR4, or irrelevant (Irr) control IgG for 1 h before infection. The presence of apoptosis was assessed by Apopercentage apoptosis Kit assay as described in the Materials and Methods. The experiment was repeated in three separate occasions. The data are presented as percent of apoptotic cells_._

### ST-11 isolates but not carriage isolates promote apoptosis of epithelial cells in MyD88-dependent manner

**Figure 2:**
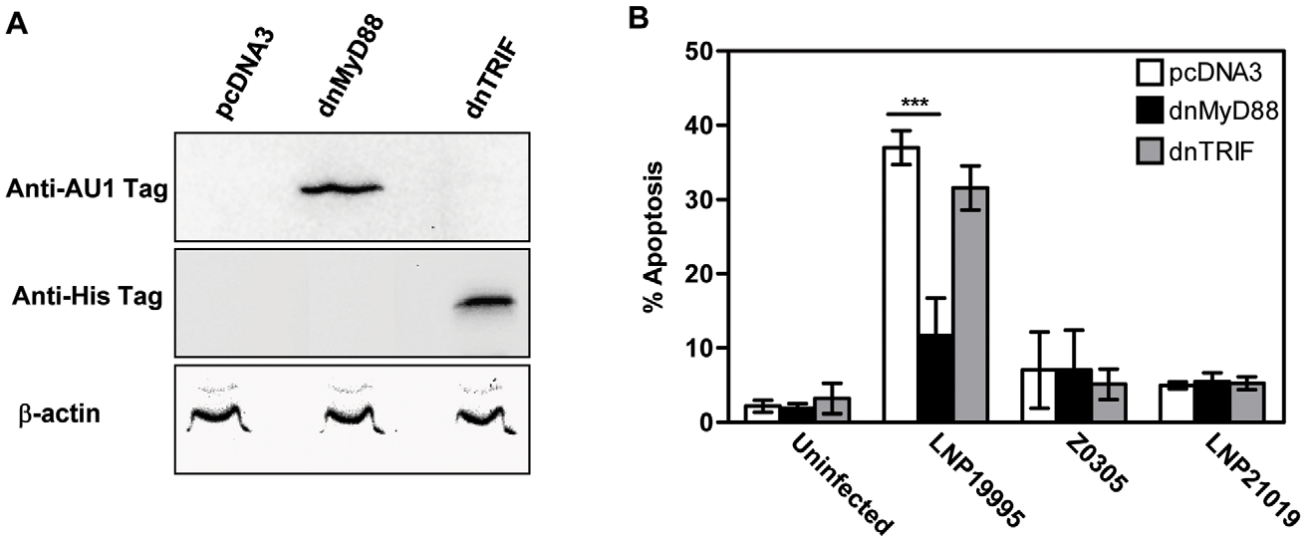
Expression of dnMyD88 but not dnTRIF inhibits ST-11-induced apoptosis. (A) Hec-1B cells were transiently transfected with either pcDNA3 empty vector or expression vector encoding either the AU1-tagged TIR domain of MyD88 (dnMyD88) or His tagged-TIR domain of TRIF (dnTRIF). Anti-AU1 and anti-His antibodies were used to confirm expression of the TIR-containing MyD88/TRIF dominant negative forms. Actin was used as loading control (B) Hec-1B cells expressing pcDNA3 vector, the dnMyD88, or dnTRIF were infected for 9 h with the indicated strains or left uninfected and assayed for apoptosis using flow cytometry. The bars represent the mean ± SD of apoptotic cells from three independent experiments. ***, *P*<0.001.

TLR4 links to both MyD88 and TRIF to transduce signals to downstream effectors [27]. MyD88 has an NH_2_-terminal death domain which links it to downstream effectors in the TLR signaling pathways and a COOH-terminal TIR domain which interacts with the cytoplasmic portion of various TLRs. Each domain expressed alone functions as a dominant negative form [28]. The TIR domain of TRIF has similar behaviour [29], [30]. To further explore the extent of LOS signaling pathway downstream TLR4 in the apoptosis promoted by the invasive ST-11 isolates, Hec-1B cells were knocked-down for functional MyD88 or TRIF by transfecting either TIR domain and apoptosis was analyzed after 9 h of infection in comparison with cells transfected with the empty vector control. Expression of AU1-tagged dnMyD88 or Myc-tagged dnTRIF was confirmed in the transfected cells by immunoblotting using specific Abs directed against each tag (Figure 2A). As expected, the apoptotic level promoted by the wild-type (WT) ST-11 isolate LNP19995 in empty vector-transfected cells was dramatically decreased after infection with the LOS-deficient isogenic mutant Z0305. Comparably to TLR4 knock-down, expression of the dnMyD88 considerably impeded apoptosis promoted by the WT ST-11 isolate LNP19995, while expression of the dnTRIF did not improve the survival of LNP19995-infected cells (Figure 2B, 11.70±5.03% of dnMyD88-transfected cells underwent apoptosis while 37.01±2.28% of pcDNA3 or 31.58±2.98% of dnTRIF cells were already apoptotic). Both transfected constructs (dnMyD88 or dnTRIF) resulted in apoptotic level in cells infected with the non invasive carriage isolate LNP21019 comparable to uninfected cells (Figure 2B). Results similar to dnMyD88 were obtained in cells transfected with a dnIRAK1, an effector protein downstream MyD88 (data not shown). Taken together, our results indicate that LOS-mediated apoptotic signaling elicited by the ST-11 isolates through TLR4 involved a MyD88- but not TRIF-dependent pathway.

### Invasive ST-11 isolates and non-invasive carriage isolates differentially modulate NF-κB activity

**Figure 3:**
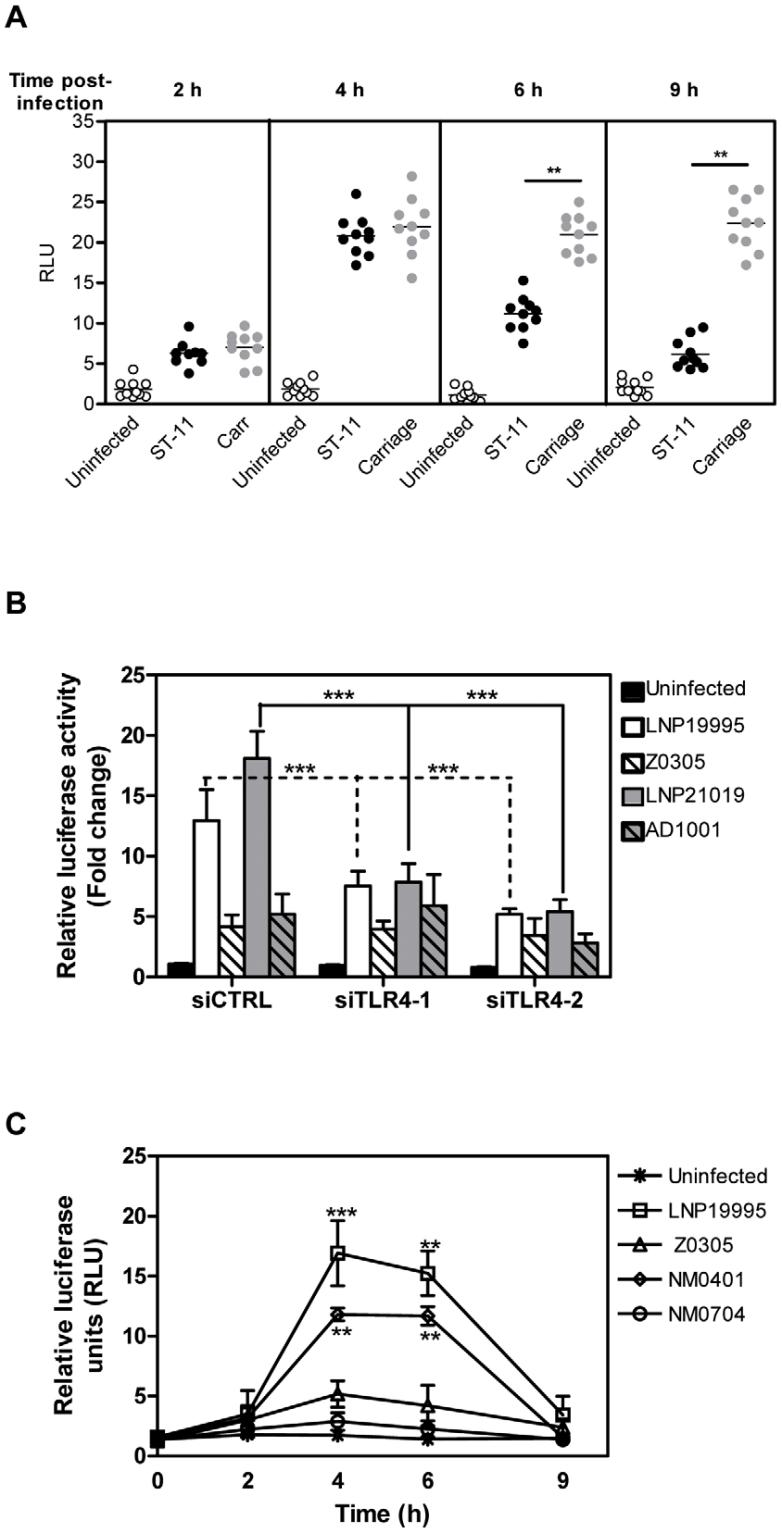
Invasive ST-11 but not carriage isolates impair NF-κB transcriptional and DNA binding activities in Hec-1B cells during the late steps of infection. (A) Kinetics of NF-κB transactivation in time course infection with the ST-11 and carriage isolates. A total of 5 x 10^4^ cells/well were transiently transfected in 96 well plates with the plasmid (Igκ)_3_conaLuc. Luciferase activities reflecting NF-κB transcriptional activities, were determined from cell lysates after each time point of infection with the ST-11 isolates (n = 10 isolates) or carriage isolates (n = 10 isolates). Transfection efficiency is normalized by co-transfection with a constitutive β-galactosidase reporter plasmid. (B) Expression of LOS and intact TLR4 signaling are required for early activation of NF-κB. Hec-B cells were co-transfected with the plasmid (Igκ)_3_conaLuc and one of the three silencing oligonucléiotides siTLR4-1 or siTLR4-2 (specific for TLR4) or siCTRL (non specific control oligonucléiotide). Transfected cells were left uninfected or infected for 4 h with the wild type strains LNP19995 or LNP21019 or the isogenic LOS-defective mutants Z0305 or AD1001, respectively. Luciferase activities were normalized by co-transfection with the plasmid pCMVβ. (C) Late alteration of NF-κB is independent on PorB expression. Hec-1B cells were transfected as described in (A) and infected with the wild type ST-11 invasive isolate LNP19995 or the isogenic mutants Z0305 (LOS^-^), NM0401 (PorB^-^) or NM0701 (LOS^-^ PorB^-^) in absence of serum. Luciferase activities were determined as described above after each time point. Data (mean ± SD) are presented as fold change of relative luciferase units (RLU) regarding uninfected cells. **, *P*<0.01 for a comparison of cells infected with the ST-11 isolates and those infected with the carriage isolates.

The subsequent steps in TLR4/MyD88 signalling lead to the activation of NF-κB which translocates into the nucleus to activate pro-inflammatory and pro-survival gene expression including TNF-α [31]. We therefore sought to determine the role of NF-κB activity in apoptosis induced by the meningococcal ST-11 isolates. We first followed the kinetic of NF-κBtrans-activation in response to meningococcal infection. For that purpose, Hec-1B cells were transiently transfected with the plasmid (Igκ)_3_conaLuc, where expression of the luciferase reporter gene is driven by an NF-κB-dependent promoter. Luciferase activity normalized to the constitutive β-galactosidase activity control was assayed in a time course infection. As depicted in Figure 3A, infection of Hec-1B cells with either isolates induced luciferase activity which peaked at 4h post-infection to nearly 20 fold relative to uninfected cells. This early activation required LOS and occurred in TLR4-dependent manner as lack of LOS from both, the invasive or the carriage isolates (Z0305 or AD1001 mutants, respectively) or silencing of TLR4 considerably reduced transactivation of NF-κB (Figure 3B). Surprisingly, NF-κB-dependent luciferase activity decreased beyond 6 h of challenge with the invasive ST-11 isolates while persisted in response to infection with the carriage isolates (Figure 3A). These data corroborate with the EMSA assays. Indeed, the DNA-binding activity of NF-κB to a specific radio-labeled probe peaked transiently at 4 h of infection then decreased beyond 6 h of infection with the ST-11 invasive isolate LNP19995, while persisted longer in cells infected with the carriage isolate LNP21019 (Supporting Figure S2). Interestingly the decrease of NF-κB-dependent luciferase and DNA-binding activities in cells infected with the ST-11 isolates was concomitant to induction of apoptosis by these isolates. Collectively, these data show a differential modulation of NF-κB-dependent transcriptional activity between the invasive ST-11 and the non-invasive carriage isolates.

LOS purified from the invasive LNP19995 or the carriage LNP21019 isolates were able to trigger a persistent transactivation of NF-κB similarly to the carriage isolates, as judged by EMSA and luciferase reporter assays. No alteration of NF-κB activity was observed at least up to 9 h of treatment with the purified LOS of both isolates (Supporting Figure S3). In absence of serum, the lack of PorB expression in the mutant NM0401 resulted in slight decrease of the early activation of NF-κB comparing to the parental ST-11 strain LNP19995. In contrast to the PorB mutant, the LOS-devoid mutant Z0305 or the mutant lacking both LOS and PorB were strongly affected (Figure 3C). Interestingly, activation of NF-κB was decreased in the later time points in cells infected with the PorB mutant NM0401 similarly to cells infected with the wild type parental strain LNP19995 (Figure 3C). Overall, our data suggest that LOS is a potent activator of NF-κB comparing to PorB. However, the late reduction of NF-κB activity provoked by the invasive ST-11 isolates seems to be independent of the expression of both PorB and LOS.

It is worth to note that expression of NF-κBp65 and p50 subunits was not affected over the time of infection with each isolates, excluding the modulation of NF-κB transcriptional activity due to the alteration of NF-κB expression (Supporting Figure S4).

### Non-invasive isolates promote sustained NF-κB activity to protect epithelial cells from apoptosis

**Figure 4:**
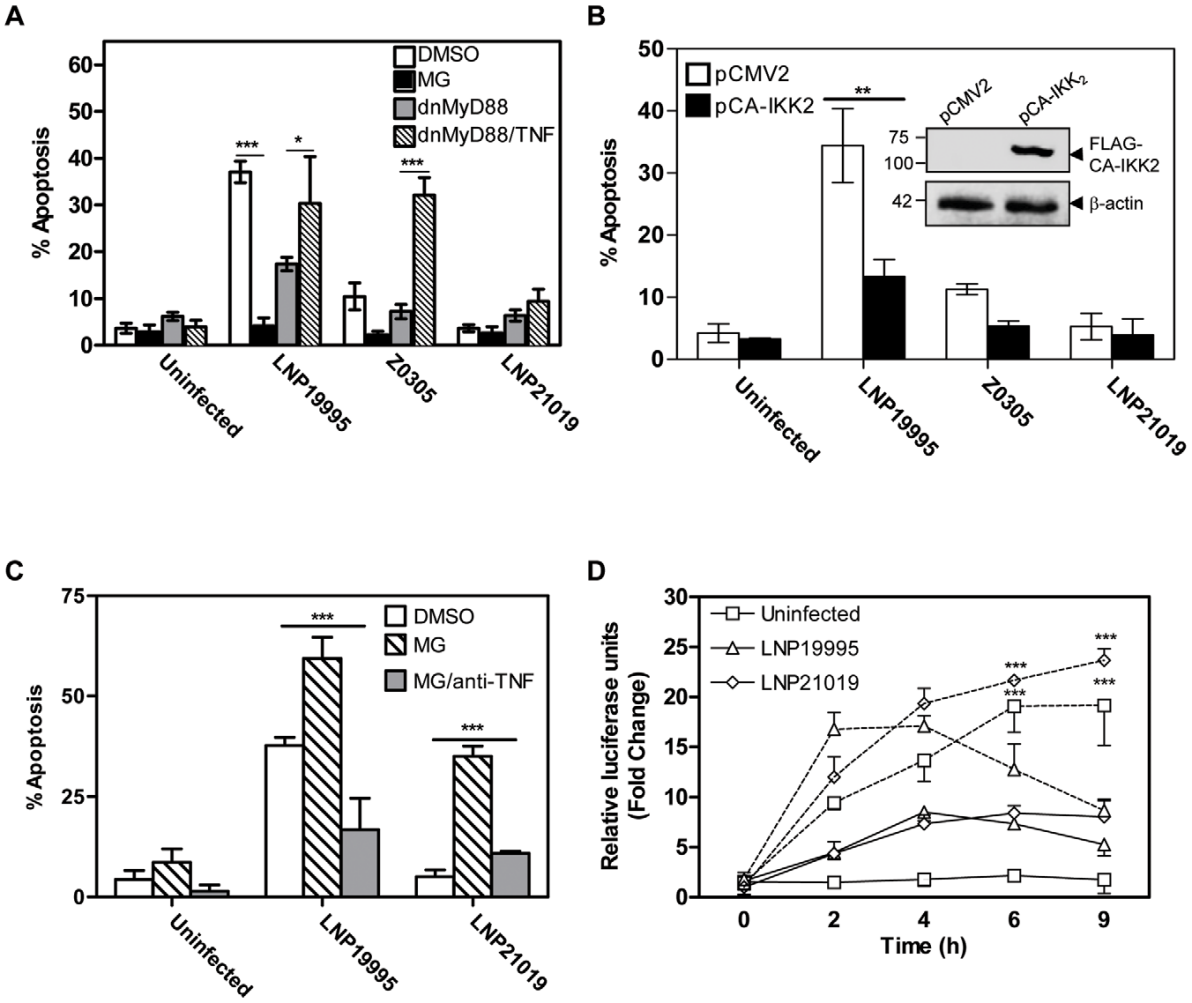
Role of NF-κB in apoptosis of Hec-1B cells induced by meningococcal ST-11 isolates. (A) Inhibition of the early activation of NF-κB abrogated apoptosis of Hec-1B cells promoted by the ST-11-isolates. Hec-1B cells were pretreated with DMSO (0.1%) or MG-132 (10 µM) for 1h, then infected with the indicated strains or left uninfected for 9 h in presence of DMSO (open bars) or MG-132 (dark bars). Similarly, cells were transfected with the dnMyD88 and infected in absence (grey bars) or presence (hatched bars) of TNF-α. Results shown are a summary of three independent experiments. ***, *P*<0.001. (B) Constitutive activation of NF-κB conferred protection of cells from apoptosis promoted by the ST-11 isolates. Hec-1B cells were transfected with the FLAG-tagged constitutive active form of IKK-2 (CA-IKK_2_) or the pCMV2 empty vector control. After transfection, cells were left uninfected or infected with the indicated strains for 9 h then analysed for apoptosis using flow cytometry. Histogram bars represent the mean ± SD from three independent experiments (C). **, *P*<0.01. Insert autoradiograph: Analysis of CA-IKK_2_ expression by immunoblot using anti-FLAG mAb. β-actin was used as loading control. Size of bands (in kDa) are indicated on the left. (C) Cells were infected as described in Materials and Methods. MG-132 alone or in presence of anti-TNF-α neutralizing mAb was added after 4 h of bacterial challenge. Apoptotic level was monitored by annexin V staining and flow cytometry. (D) Cells were co-transfected with the plasmid (Igκ)_3_conaLuc and the plasmid carrying dnMyD88. After transfection, cells were infected with the indicated strains for 9 h in absence (solid lines) or presence (dashed lines) of TNF-α. Luciferase activities were determined after each time point as indicated before. Data are mean ± SD from three independent experiments.

To further explore the role of NF-κB in the apoptosis induced by the meningococcal ST-11 isolates, early activation of NF-κB was blocked 1 h prior to infection, using MG-132 inhibitor. MG-132 is a peptide-aldehyde protease inhibitor that blocks NF-κB activation via inhibition of the proteasome function [32], [33]. Hec-1B cells were then infected for 9 h in presence of a non-toxic concentration of MG-132 or the carrier solvent DMSO (0.1%). Neither MG-132 nor DMSO alone resulted in apoptosis above background levels in uninfected cells (data not shown). Unexpectedly, pre-treatment of cells with MG-132 effectively protected cells from apoptosis brought about infection with the invasive isolate comparing to cells infected in presence of DMSO (Figure 4A). Similar results were obtained using the specific NF-κB inhibitor IKK-Nemo Binding Domain (NBD) peptide (data not shown). Early activation of NF-κB seems then to be a pre-requisite to promote apoptosis of epithelial cells with the invasive isolates. This requirement contrasts the cytoprotective role of NF-κB reported by several groups [34]–[38]. Nevertheless, the kinetic of NF-κB transactivation reported in Figure 3A showed that both invasive and non-invasive isolates are able to promote NF-κB activity after 4h of infection. However, only invasive ST-11 isolates are able to induce apoptosis after 9h of infection that was correlated with reduced NF-κB activity. Non-invasive isolates promoted sustained NF-κB activity that correlated with protection against apoptosis (Figure 3). We therefore explored the extent of the late down-regulation of NF-κB activity on the apoptotic cell death induced by the ST-11 invasive isolates. Hec-1B cells were transfected with a FLAG tagged-constitutively active form of IKK_2_ (CA-IKK_2_), leading to a constitutive phosphorylation and degradation of IκBα and subsequent increase of NF-κB activity, [39] or pCMV2 empty vector control (Figure 4B, immunoblot insert). Expression of CA-IKK_2_ was able to abrogate LNP19995-induced apoptosis by four fold comparing to empty vector-transfected cells (Figure 4B). These results strongly suggest that the late down regulation of NF-κB activity is required for invasive ST-11 isolates to promote LOS-mediated apoptosis of epithelial cells.

One possible explanation for this dual effect of NF-κB activation is that early activation of NF-κB is required to promote expression of TNF-α, which acts lately to promote apoptosis of epithelial cells following the decrease of NF-κB activity. Indeed, MG-132 dramatically reduced the level of secreted TNF-α with respect to DMSO-treated cells (Supporting Figure S5). To test this hypothesis, MG-132 was incorporated after 4 h of bacterial challenge (the period of which NF-κB transactivation was peaked). In these conditions, apoptosis was strongly induced irrespective to infection with the invasive or the carriage isolates (Figure 4C). The presence of anti-TNF-α neutralizing antibody significantly abrogated this effect (Figure 4C). To further analyse the role of TNF-α in meningococcal induced apoptosis, TLR4-mediated NF-κB activation was blocked by transfecting cells with the dnMyD88-expressing vector. Indeed, expression of the dnMyD88 as MG-132 pre-treatment strongly reduced the levels of secreted TNF-α in cells infected with both the invasive or the carriage isolates when compared to cells transfected with the empty vector control (Supporting Figure S5). The apoptotic level promoted by the invasive isolate LNP19995 also decreased significantly in cells transfected with the dnMyD88 (Figure 4A). Addition of TNF-α rescued apoptosis regarding cells transfected with the dnMyD88 and infected in absence of TNF-α. In all tested conditions, the carriage isolate induced low apoptosis similar to the background level (Figure 4A). These results underline the key role of TNF-α in promoting apoptosis by the invasive, but not the carriage isolates. In contrast to LOS, TNF-α also activates NF-κB but in MyD88-independent manner [40] and Figure 4D. After 9 h of incubation, TNF-α-induced NF-κB activity was also deceased upon infection with the invasive isolate LNP19995 but not with the carriage isolate LNP21019. Collectively, these data suggest that TNF-α secreted early upon infection with the ST-11 isolates is required to sensitize cells to apoptosis in synergy with the late down-regulation of NF-κB transcriptional activity.

### Increased shedding of TNFR1 in cells infected with non-invasive isolates occurs in TACE/ADAM17-dependent manner

**Figure 5:**
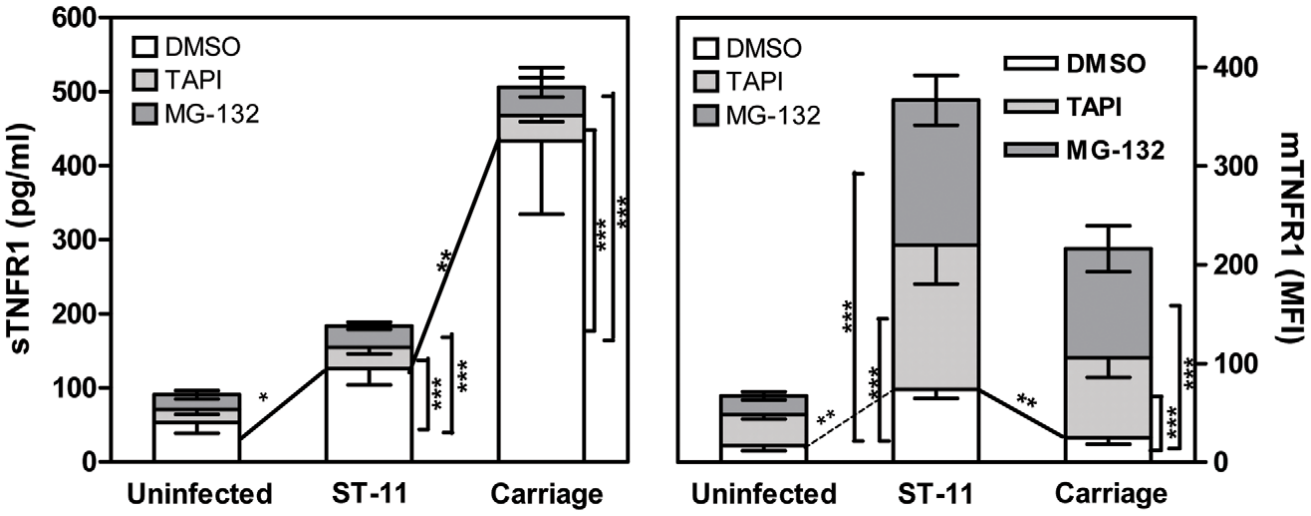
Role of TACE/ADAM17 and NF-κB activation in cell surface expression and shedding of TNFR1. Hec-1B cells were either pre-treated with DMSO (open bars), TAPI-1 (dotted bars) or MG-132 (grey bars) and infected with the invasive ST-11 isolates (n = 10) or non invasive carriage isolates (n = 10) or left uninfected for 9 h. Supernatants were then collected, cleared from bacteria and assayed for sTNFR1 in specific ELISA using anti-sTNFR1 mAb (*left panel*). Commercialized sTNFR1 was used as standard. Cells were also harvested and surface stained using anti-TNFR1 mAb and FITC-conjugated goat anti-mouse IgG Ab (*right panel*). Data are expressed as mean fluorescence intensity (MFI). Values are means ± SD of two independent experiments, each strain was assayed in triplicate. For statistical analysis, TAPI- or MG-132-treated samples were compared to DMSO-treated samples within each group of strains (ST-11 or carriage; ***, *P*<0.001). ST-11-infected *vs.* carriage-infected cells were also compared within each treatment (**, *P*<0.01).

We have previously shown that carriage non-invasive isolates inhibit TNF-α-dependent apoptotic pathway through increasing the shedding of soluble TNFR1 (sTNFR1) which interfere with the biological activity of TNF-α. Increased shedding of sTNFR1 occurred concomitantly to the decreased level of the membrane-associated TNFR1 (mTNFR1) [22]. Shedding of TNFR1 is mediated by TNF-α converting enzyme (TACE also known as ADAM17), a metalloproteinase localized to the cytoplasmic membrane [41], [42]. One possible explanation for the increased shedding of sTNFR1 in cells infected with the carriage isolates could be the increased activity of TACE/ADAM17. We therefore analyzed the ability of the specific TACE/ADAM17 inhibitor, TNF-α protease inhibitor–1 (TAPI-1), to block TNFR1 release in response to infection with the carriage isolates. At the concentration employed (1 µM), TAPI-1 did not compromise cell viability as judged by PI staining (data not shown). As depicted in Figure 5, cells infected for 9 h with the carriage isolates in presence of TAPI-1 failed to induce shedding of sTNFR1 comparing to DMSO-treated cells (Figure 5, left panel; 34.56±7.37 pg/ml for TAPI-treated cells *vs.* 433.3±58.75 pg/ml for DMSO-treated cells, *P*<0.001) and concomitantly displayed significant surface levels of membrane bound-TNFR1 (Figure 5, right panel; MFI 81.12±15.49 in TAPI-treated cells *vs.* 24.94±4.15 in DMSO-treated cells, *P*<0.001). TAPI-1 treatment further increased mTNFR1 level in cells infected with the ST-11 invasive isolates comparing to cells infected in presence of DMSO (Figure 5, right panel; MFI 146.4±35.79 *vs.* 73.88±5.48, *P*<0.001). These data were also confirmed by immunofluorescence microscopy examination (Supporting Figure S6). These results suggest that increased shedding of sTNFR1 in cells infected with the carriage non-invasive isolates involved TACE/ADAM17 activity.

### Increased TACE/ADAM17-mediated shedding of TNFR1 depends on sustained NF-κB activation

**Figure 6:**
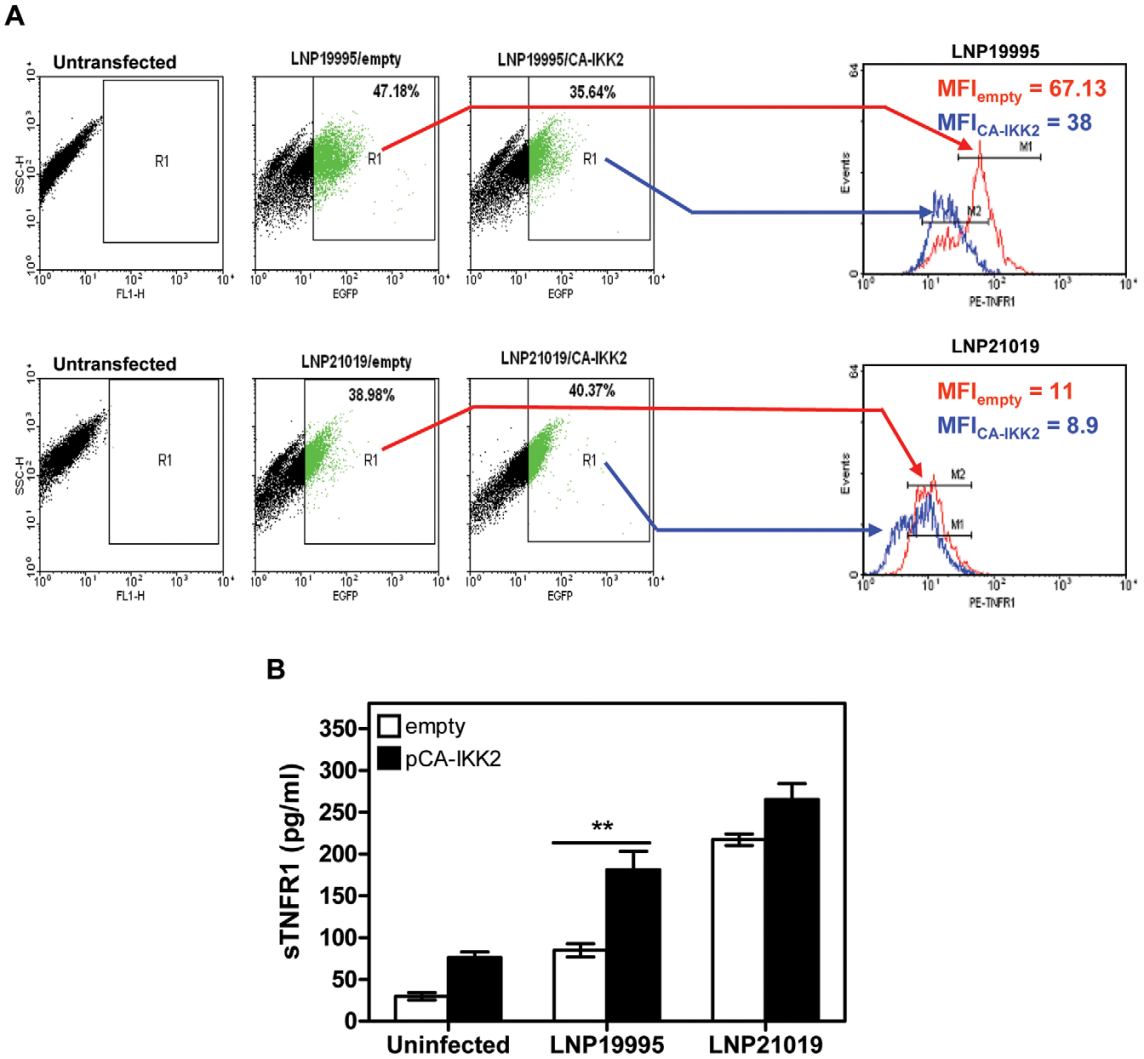
Constitutive activation of NF-κB decreased surface expression of TNFR1 and increased sTNFR1 shedding. (A) Analysis of surface expression of TNFR1. FACS analysis of GFP-transfeted cells. Hec-1B cells were transfected with the pCA-IKK_2_-GFP (in blue) or empty vector control pCMV-GFP (in red). Cells were then infected with either LNP19995 or LNP21019. After infection cells were surface-stained for TNFR1 using specific anti-TNFR1 mAb followed by phycoerythryn (PE)-conjugated secondary Ab. Dot plots in the *left panel* show the percentages of GFP+ subpopulation (FL1 channel) within the total cells incubated with LNP19995 or LNP21019 relative to untransfected cells. GFP^+^ events were gated as region R1 and surface expression of TNFR1 (FL2 channel) was analyzed within this GFP^+^ subpopulation. Histogram plots (*right panels*) show the level of TNFR1 at the surface of LNP19995 infected or LNP21019-infected cells. TNFR1 levels were expressed as the mean fluorescence intensities (MFI) relative to unstained cells. The histograms corresponding to each gated region is indicated by arrows. (B) sTNFR1 was assayed by ELISA after exposure of pCA-IKK_2_-(closed bars) or empty vector-transfected (open bars) Hec-1B cells to LNP19995 or LNP21019. Data represent the mean ± S.D. from three independent experiments, each performed in duplicates. **, *P*<0.01 of CA-IKK_2_-transfected cells compared with empty vector control-transfected cells.

Given that carriage and invasive ST-11 isolates differentially modulate the late activity of NF-κB, we sought to determine whether the modulation of NF-κB transcriptional activity may impact the surface display and extracellular shedding of TNFR1. To address this issue, MG-132 was added after 4 h of bacterial challenge to not compromise the early expression of TNF-α. Comparing to DMSO vehicle-treated cells, MG-132-treatment led to significant decrease of sTNFR1 shedding and increase of mTNFR1 level in cells infected with the carriage isolates (Figure 5). To test whether persistent transcriptional activity of NF-κB would inverse the surface display of TNFR1 due to infection with the ST-11 isolates, Hec-1B cells were transiently transfected with the plasmid pCA-IKK_2_ that was modified by insertion of EGFP marker to visualize transfected cells. As control, cells were transfected with pcDNA3 harbouring the same marker (empty vector). As shown in Figure 6A, 39-47% and 35-40% of cells were transfected with empty vector control and pCA-IKK_2_, respectively (*left panels*). After 9 h of infection, surface expression of TNFR1 (mTNFR1) was examined in this sub-population of transfected cells (GFP+ events gated in region R1, Figure 6A, *left panels*). As expected, among empty vector-transfected cells, infection with the ST-11 isolate LNP19995 resulted in higher level of mTNFR1 compared to cells infected with the carriage isolate LNP21019 (MFI 67.13 *vs.* MFI 11, respectively). Interestingly, among pCA-IKK_2_-transfected cells, infection with the ST-11 isolate LNP19995 led to almost 50% decrease of TNFR1 surface level compared to empty vector-transfected cells (Figure 6A, *right panels*). Consistent with these results, the level of sTNFR1 significantly increased in CA-IKK_2_ transfected-, LNP19995-infected cells compared to empty vector-transfected, LNP19995 infected cells or cells infected with the carriage isolate LNP21019 (Figure 6B). Taken together, these results suggest that the differential modulation of NF-κB activity between the invasive ST-11 and the non invasive carriage isolates resulted in differential surface display of TNFR1 in TACE/ADAM17-dependent way.

### Apoptosis of epithelial cells promoted by ST-11 isolates involves a sustained JNK activation

**Figure 7:**
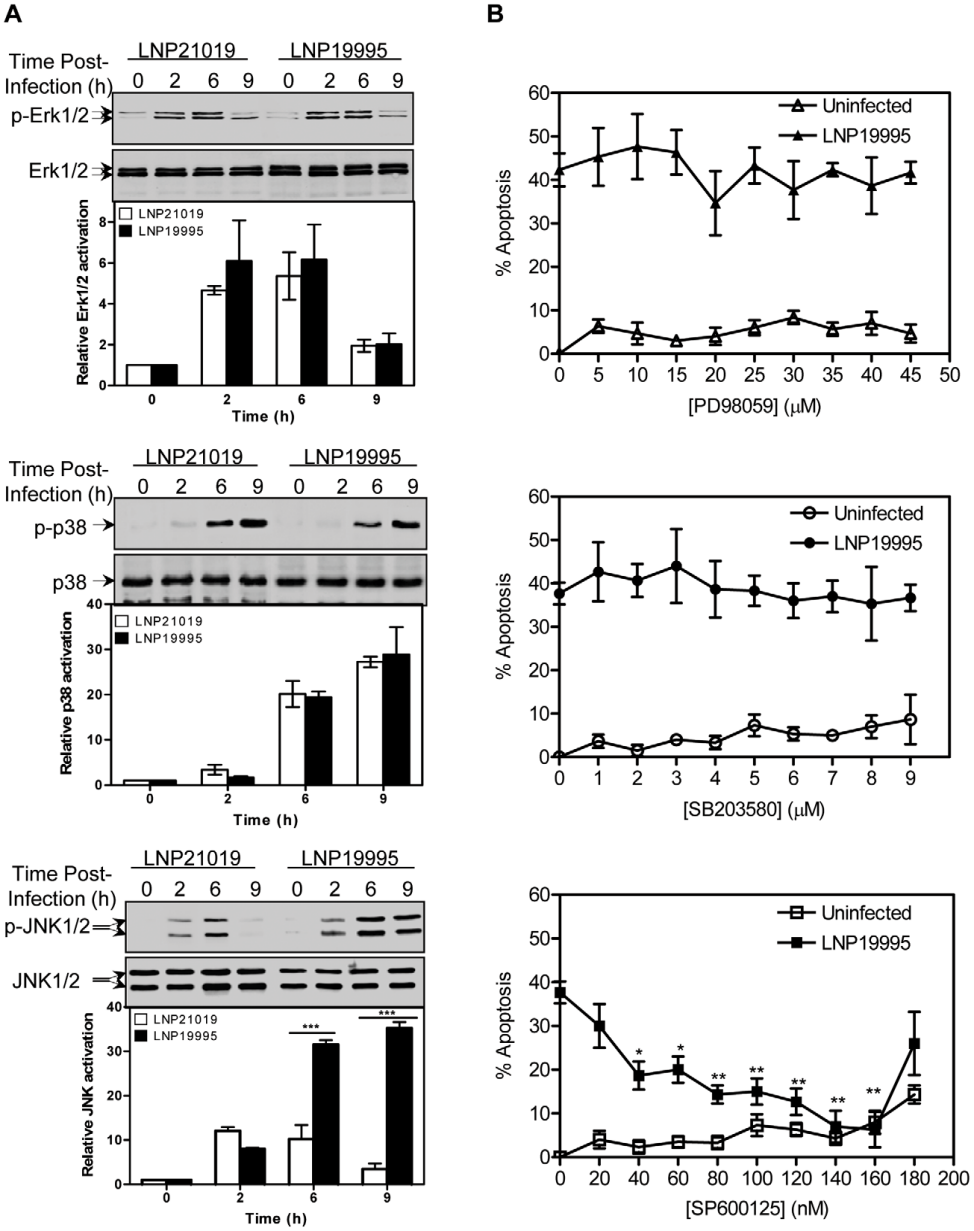
Role of MAPK in apoptosis of Hec-1B cells induced by the ST-11 isolates. (A) Effect of ST-11 and carriage isolates on expression and phosphorylation of MAPK proteins. Hec-1B cells were infected with the indicated isolates for the indicated time points. Samples were lysed and immunoblotted with rabbit polyclonal anti-phospho-p44/42 (ERK1/2) (*upper panel*), anti-phospho-p38 (*middle panel*), anti-phospho-JNK (*lower panel*) antibodies. The membranes were subsequently stripped and reprobed with rabbit polyclonal anti-ERK, p38 or JNK antibodies as protein loading controls. Blots are representative of three separate experiments with similar results. Histograms below each blot represent the mean values from densitometry scans, adjusted with total MAPK, and expressed as fold changes relative to t = 0 h set to 1. (B) Effect of MAPK inhibitors on the LNP19995-induced apoptosis of Hec-1B cells. The cells were pretreated with different concentrations of inhibitors of ERK phosphorylation (PD98059, *upper panel*), p38 phosphorylation (SB203580, *middle panel*), or JNK phosphorylation (SP600125, *lower panel*) for 1 h before infection or not with LNP19995 for 9h. Apoptosis was measured using Apopercentage apoptosis assay kit. Means ± SD of two independent experiments, each performed in triplicates, are shown. *, *P*<0.05; **, *P*<0.01 *versus* LNP19995 alone without inhibitor.

MAP kinase (MAPK) signaling pathways are activated in inflammatory reactions and have been shown to play important role in cell growth and death [43]. In general, p38 and c-Jun N-terminal kinase (JNK) are involved in cell death mechanisms, whereas Extracellular signal-regulated kinase (Erk1/2) is critical for cell survival [44]. We therefore investigated the potential role of MAP kinases in apoptosis of epithelial cells induced by the pathogenic meningococcal isolates. First, the effect of meningococci on the activation of the above mentioned kinases was examined in time course infection using phospho-specific antibodies. We monitored for total levels of each kinase to ensure that any change in phosphorylated protein levels was an actual measure of activation. Phosphorylation of Erk1/2 increased after 2h of cell exposure to each meningococcal isolate and then decreased after 6 h (Figure 7A, upper panel). Phosphorylation of p38 MAP kinase increased beyond 6 h of infection and persisted thereafter (Figure 7A, middle panel). Interestingly, phosphorylation of JNK increased gradually up to 6 h post infection, and then decreased afterwards to almost reach the basal level in cells infected with the carriage non invasive isolate. At the opposite, JNK phosphorylation was sustained in cells infected with the ST-11 isolate LNP19995 (Figure 7A, lower panel). As the carriage isolate, JNK was transiently activated upon treatment of Hec-1B cells with the LOS purified from the invasive or the carriage isolates (Supporting Figure S3). These data corroborate with the sustained activation of NF-κB. In all tested conditions, the total expression of each kinase was not altered (Figure 7A). To examine more thoroughly the role of the prolonged JNK activation in the cell death promoted by the invasive ST-11 isolates, we determined the effects of a dose range of the specific JNK inhibitor SP600125 [45] on apoptotic cell death induced by the invasive isolate LNP19995. Pre-treatment of cells with SP600125 markedly subdued the extent of the invasive isolate LNP19995-mediated cell death in dose-dependent manner with a maximum effective dose of 140 nM (Figure 7B, lower panel). This dose had no significant effect on viability of uninfected cells. In contrast to JNK, inhibition of p38 MAPK and Erk1/2 phosphorylation with their respective specific inhibitors had no beneficial effect on LNP19995-induced epithelial cell death and no effect on the viability of uninfected cells (Figure 7B, middle and upper panels, respectively). These data pointed out a selective differential ability of isolates to modulate the activation of JNK and suggest that infection with ST-11 invasive isolates induced death signals involving JNK, while attenuating survival signals in epithelial cells.

### Sustained JNK activation promoted by ST-11 isolates is associated with reduction of NF-κB activity and occurs in TNFR1-dependent manner

**Figure 8:**
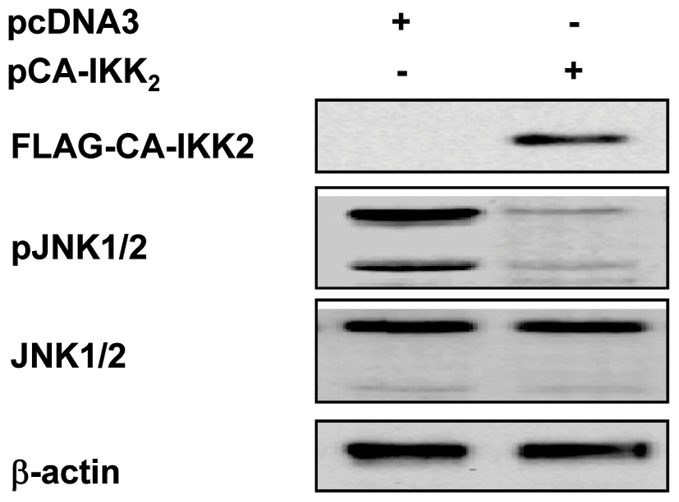
Constitutive activation of NF-κB compromised activation of JNK induced by the ST-11 isolates. Hec-1B cells were transfected with FLAG-tagged CA-IKK_2_ or empty vector control and infected for 9 h with LNP19995 isolate. After infection, cells were harvested and controlled for the expression of FLAG-tagged CA-IKK_2_ by immunoblotting using specific anti-FLAG mAb as well as for the activation of JNK using phophospecific rabbit polyclonal antibody. As loading control, total JNK and actin were analysed by specific rabbit polyclonal and monoclonal antibodies, respectively. The blot is a representative of three independent experiments which yielded similar results.

Previous observations reported that sustained JNK activation associated with inhibition of NF-κB activation, contributes to TNF-α-induced apoptosis [46], [47]. To determine the extent of the late reduction of NF-κB activity mediated by the ST-11 isolates on the sustained JNK activation, Hec-1B cells were transiently transfected with the CA-IKK_2_ and the activation of JNK was analyzed after 9 h of infection with the invasive isolate LNP19995. Comparing to the control empty vector-transfected cells, maintenance of NF-κB activation in CA-IKK_2_-transfected cells resulted in dramatic impairment of JNK phosphorylation (Figure 8) that was associated with improvement of cell survival (Figure 4C). Our data establish a direct cause-effect of interference of ST-11 invasive isolates with NF-κB activity to allow a sustained JNK activation that is necessary to promote apoptosis of epithelial cells.

## Discussion

Isolates of several bacterial species such as *N. meningitidis* may exist as symbiote in their host but may also invade internal compartments of the host with important local and systemic inflammatory responses. The consequences of the interaction with epithelial cells at the nasopharynx (the portal of entry of *N. meningitidis*) are therefore crucial in the pathophysiology of meningococcal infections. Induction of cytokines and particularly TNF-α has been implicated in local disruption of epithelial barrier functions [48], [49] and inducing epithelial cell apoptosis [50]. This hallmark may pave the way for further meningococcal invasion and dissemination to deeper sites (septicaemia and meningitis) [19]. Indeed, it has been reported that patients with meningococcal sepsis or meningitis often describe signs of pharyngitis before the onset of invasive disease while carriage isolates persist in the pharynx asymptomatically [51]. We have previously reported the impressive ability of the invasive ST-11 meningococcal isolates to induce apoptosis of epithelial cells. This overwhelming process, driven in part by the major bacterial endotoxin LOS, is dependent on an autocrin mechanism of TNF-α signaling through its receptor TNFR1 [22]. In contrast, infection with the non-invasive carriage isolates is associated with protection of epithelial cells mediated by the shedding of sTNFR1 resulting in alteration of the biological activity of TNF-α through soluble receptor-ligand complex formation and abrogation of apoptosis [22]. In this work, we further identified the actors in the signalling pathways leading to this different behaviour of invasive and non invasive isolates.

Our data with *si*TLR4 approach confirm the role of TLR4 as a potential transducer of the LOS induction of apoptotic signalling as in other Gram-negative pathogens such as *Yersinia* and *Salmonella* [52], [53]. Our data further show that TLR4 signaling induced by *N. meningitidis* through occurs in MyD88-dependent manner [54], [55], but not through the TRIF adaptor molecule (MyD88-independent pathway) [54], [56]. Indeed, these results are in agreement with the previous reports showing that MyD88 and IRAK1 are involved in apoptotic signaling upon stimulation with bacteria or bacterial components [57], [58]. TRIF-dependent pathway (MyD88-independent pathway) is more involved in generating a type I IFN-dependent response that is essential to host defence against viral infection [54], [56]. Nevertheless, many TLRs signal through the adaptor protein MyD88. In this regard, our data cannot exclude the role of other TLRs (which also signal through MyD88 such as TLR2) in the regulation of apoptosis upon infection with the pathogenic meningococcal isolates. Expression of dn-MyD88 as TLR4 knock-down, considerably altered the apoptosis induced by the ST-11 isolates and dramatically reduced expression of TNF-α. Moreover, our data suggest that the initial TLR4-dependent activation of NF-κB is required to establish apoptosis most likely through induction of TNF-αexpression. Treatment with MG-132 after 4 h of infection promoted apoptosis in cells infected with the carriage isolates and further increased apoptosis induced by the ST-11 isolates. However, sustained NF- κB activity seems to be protective against apoptosis as constitutive NF-κB activation mediated by the CA-IKK_2_, rescued the viability of the ST-11-infected cells. Interference with the NF-κB transcriptional activity to promote apoptosis of host cells has been described for other pathogens. Uropathogenic *E. coli* (UPEC) was able to abrogate urothelial responses by blocking NF-κB translocation to the nucleus and by inhibiting NF-κB-dependent transcription in response to either LPS or TNF-α stimulation [59]. *Yersinia* induces apoptotic cell death in macrophages by type III secretion system-mediated suppression of NF-κB activation [60]. In our hands, expression level of NF-κB p65 and p50 subunits were not affected during meningococcal infection, excluding the possibility that invasive ST-11 isolates modulate the activity of NF-κB through alteration of its expression. It has been shown that PorB activates NF-κB in TLR2-dependent manner [61]. The late reduction of NF-κB activity seems to be independent on PorB expression although the early activation slightly decreased comparing to the wild type strain. These results comfort our previous results showing that ST-11 isolates induce apoptosis in two independent pathways an extrinsic pathway promoted by LOS and an intrinsic pathway promoted by PorB [22]. How ST-11 meningococcal isolates attenuate the activity of NF-κB to promote apoptosis of epithelial cells is currently under investigation. Bacterial factor(s) may be responsible for this difference in the fate of NF- κB activity upon meningococcal infection.

As a biological consequence of the differential modulation of NF-κB activity between ST-11 and carriage isolates, cells exhibited: i) a differential display of TNFR1 expression at the surface of infected cells dependent on TACE/ADAM17 activity (higher mTNFR1 and low sTNFR1 in cells infected with the ST-11 isolates versus low mTNFR1 and higher sTNFR1 in cells infected with the carriage isolates) and ii) a differential profile of JNK activation (sustained activation in cells infected with the ST-11 invasive isolates and transient activation in cells infected with the carriage non invasive isolates). Indeed, we demonstrated that the inducible shedding of TNFR1 from cells infected with carriage isolates can be blocked by TAPI-1, an inhibitor of TACE/ADAM17. Furthermore NF-κB inhibitor suppressed the shedding of TNFR1 in carriage isolates infected cells while constitutive activation of NF-κB resulted in increased TNFR1 shedding from ST-11-infected cells. Our data are in agreement that sustained NF-κB activity is associated with up-regulation, maturation and increased activity of TACE/ADAM17/ADAM17 [62]. Nevertheless, the precise mechanism by which the meningococcal isolates modulate TACE/ADAM17 activity has yet to be identified. Recently, the involvement of the MAPK pathway in response to infection by *N. meningitidis* has been reported. It has been demonstrated that *N. meningitidis* can induce a sustained activation of p38 MAPK and JNK *in vitro* [63]. However, the role of these MAPKs in meningococcal-induced cell death has not been documented. It has been suggested that p38 and JNK are in general involved in cell death mechanisms, whereas Erk1/2 is critical for cell survival [64]. Our findings provide evidence for the participation of sustained JNK phosphorylation in the regulation of epithelial cell apoptosis in response to infection by ST-11 meningococcal invasive isolates. The JNK activity was only transiently detected with non invasive isolates. Using microarray analysis, we have recently reported that TACE/ADAM17 expression is reduced in blood of infected mice with *N. meningitidis* ST-11 (- 2.4 fold change and Z score  =  -1.7) [65]. On the other hand, it has been shown that TACE/ADAM17 activity increases upon loss of c-Jun, a downstream target of JNK [66]. Sustained JNK activation promoted by ST-11 isolates may therefore be involved in the increase of TNFR1 surface expression through activation of c-Jun.

**Figure 9:**
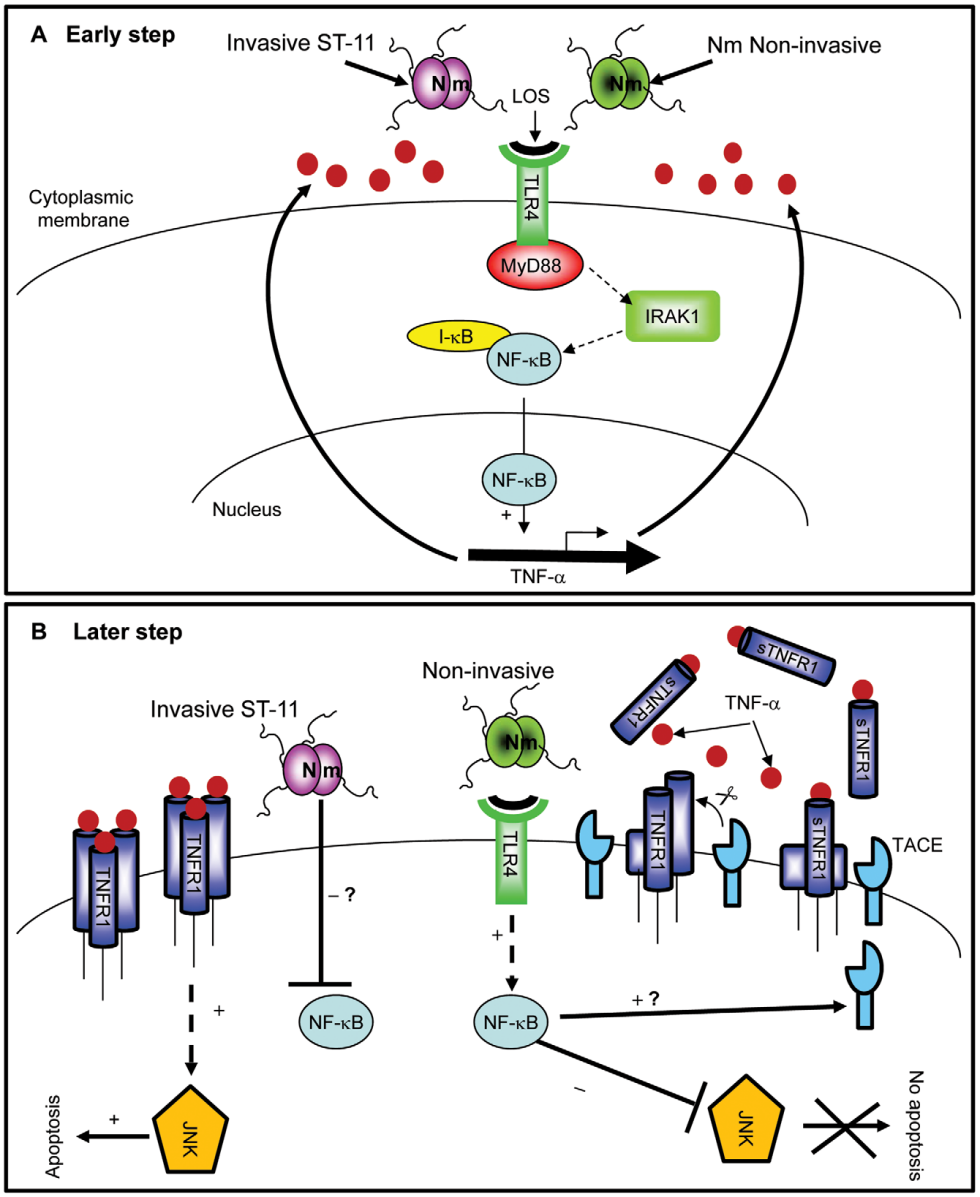
Differential modulation of NF-κB activity by invasive ST-11 and non-invasive carriage isolates leading to cell death and survival of epithelial cells. (A) Early during infection, LOS recognition with TLR4 leads to activation of NF-κB in MyD88/IRAK1-dependent manner. Activation and nuclear translocation of NF-κB induces expression and secretion of TNF-α. (B) Persistent NF-κB activity in cells infected with non-invasive isolates antagonizes activation of JNK and induces TACE activity with a yet unidentified mechanism. Induced TACE activity would be responsible for increased shedding of sTNFR1 which interferes with TNF-α apoptotic signaling and protects cells against apoptosis. In contrast, later attenuation of NF-κB activity by ST-11 invasive isolates with an unknown mechanism leading to activation of JNK-dependent apoptotic cell death.

Based on these results, our data lean toward a biphasic model to promote apoptosis by ST-11 isolates (Figure 9): First, LOS mediates early activation of NF-κB during meningococcal infection in TLR4/MyD88/IRAK1-dependent manner leading to induction of expression and early secretion of the pro-inflammatory cytokine TNF-α and its receptor TNFR1 (Figure 9A). The sustained NF- κB activity may then promote TACE/ADAM17 activity that allows shedding of TNFR1 and prevents a sustained activation of the apoptotic factor JNK. This may then protect epithelial cells against apoptosis when they encounter non invasive carriage isolates. At the opposite, invasive isolates may produce factor(s) that inhibit the NF- κB activity leading to the accumulation of membrane bound TNFR1 and a sustained activation of the apoptotic factor JNK. (Figure 9B).

*N. meningitidis*-host cell interaction seems to involve a complex process in which bacterial heterogeneity impact differentially on the modulation of host cell signaling. Knowledge of the mechanism of alteration of NF-κB activity related to the detrimental effect of ST-11 invasive isolates may therefore provide better understanding and rational approaches for the control of invasive meningococcal infection.

## Materials and Methods

### Reagents and antibodies

RPMI 1640, HBSS and trypsin-EDTA were obtained from Invitrogen (France). Cocktail of protease inhibitors and phenylmethylsulfonyl fluoride (PMSF) were from Boehringer Mannheim (France). Human TNF-α (hTNF-α) was from Clinisciences (France). MG-132, SP600125, SB203580 and PD98059 were obtained from Sigma-Aldrich. Firefly D-luciferin was purchased from Caliper (France). Mouse anti-AU1, anti-Myc, anti-FLAG, anti-HA, anti-hTNFR1 mAbs and anti-p65 and anti-p50 rabbit polyclonal antibodies were purchased from abcam (France). human anti-TLR4 and anti-actin mAbs were from sigma-aldrich. Neutralizing anti-TLR4 mAb (clone HTA125) was from eBioscience (Hatfield, UK). Rabbit antibodies directed against JNK, p38 MAPK, Erk1/2, and their phosphorylated forms were obtained from Cell signaling. Horseradish peroxidase (HRP)-conjugated IgG antibodies were obtained from Jackson ImmunoResearch. FITC- and Texas Red-conjugated secondary antibodies were obtained from Invitrogen.

### Plasmid constructs and siRNA oligonucleiotides

The AU1-MyD88_152-296_ (dnMyD88) and MycHis-IRAK1_1-215_ (dnIRAK1) constructs were a generous gift from Dr. Muzio Marta (San Raffaele Scientific Institute, Division of Molecular Oncology, Milano, Italy). The pSH241 plasmid [67] carrying the TIR domain of TRIF (pcDNA-TRIF-TIR_397-530_, dnTRIF) was kindly provided by Dr. Wilbert A. Derbigny (Department of Microbiology and Immunology, Indiana University, School of Medicine). The (Igκ)_3_conaLuc plasmid harbouring NF-κB-dependent luciferase reporter construct was kindly provided from the Dr. Alain Israel's laboratory (Institut Pasteur, Unité de signalisation moléculaire et Activation cellulaire). The β-galactosidase expression plasmid pCMVβ was a generous gift from Dr. Laurence Arbibe, (Institut Pasteur, Unité de Pathogénie Microbienne Moléculaire). The plasmid IKK-2 S177E S181E carrying the FLAG-tagged constitutively active form IKK-2 (pCA-IKK_2_) (due to two point mutations in the S177E and S181E [39]) was purchased from Addgen (Cambridge, MA). To insert the EGFP into pCA-IKK_2_, the EGFP cassette under the control of the CMV promoter was obtained by digesting the plasmid pEGFP-C1 (Clontech) using *Ase*I and *Xho*I restriction enzymes. The gel-purified cassette was then blunt-ended using the Klenow fragment of DNA polymerase (Fermentas) and was inserted into the blunt-ended *Age*I restriction site of pCA-IKK_2_ plasmid. This recombinant plasmid was named pCA-IKK_2_-GFP.. pcDNA3 and pCMV2 empty vectors were respectively from Invitrogen and Addgen. All plasmid DNAs were prepared with a Endofree Maxi prep kit (Qiagen). *si*RNA oligonucléiotides used in this study are listed in the Table S1 and were purchased from Sigma proligo (France).

### Bacterial strains and growth conditions

*Escherichia coli* DH5α strain [68] was used for plasmid propagations. DH5α was grown in Luria- Bertani (LB) medium supplemented with 100 µg/ml ampicilin. Meningococcal clinical isolates in France are sent to the National Reference Centre for Meningococci (NRCM) for full determination and typing. Bacteria were grown in GCB medium with Kellogg supplements [69]. Phenotypes (serogroup: serotype: serosubtype) and MLST genotypes were determined as previously described [9]. Sequence types (ST) and clonal complexes were assigned using the *Neisseria* MLST database (http://pubmlst.org/neisseria). All *N. meningitidis* strains used in this study were previously characterized [22] and their characteristics are listed in Supporting Table S2. The mutant strains NM0401, Z0305 and NM0701 were described previously [22]. The strain AD1001 inactivated in *lpxA* gene involved in LOS biosynthesis was generated by transforming the genomic DNA of Z0305 strain (*lpxA::aph3'*) in the parental strain LNP21019. Positive clones were selected on GCB agar plates supplemented with 100 µg/ml kanamycin. Knock-out mutant was further confirmed by PCR and Southern blot.

### Cell culture, transient transfection, short interfering RNA silencing (siRNA) and infection

Human endometrial epithelial cell line (Hec-1B) has been widely used as a model for meningococcal infections [70], [71]. This cell line express a surface CD14 and TNFR1 and secrete TNF-α upon infection [22], [72]. These features make it therefore suitable for this study. Hec-1B cells were maintained in RPMI-1640 (Invitrogen, France) supplemented with 5% foetal bovine serum (Sigma), 50 U/ml penicillin and 50 µg/ml streptomycin. Hec-1B cells were washed extensively and harvested by Trypsin-EDTA. Depending to experiments, cells were plated in 10 cm-culture dish, or seeded in 12- or 96-well culture plates (Costar) at a density of ∼ 5×10^5^ per cm^2^. Transfection experiments were carried-out prior to infection with 3* µ*g of plasmid DNA or 25 nM *si*RNA (Sigma) using Lipofectamine 2000 (Invitrogen) according to the manufacturer's instructions. All transfection experiments were performed in FBS- and antibiotics-free medium. Forty eight hours post-transfection, cells were washed and infected in absence of antibiotics and in presence of serum (unless otherwise indicated) with bacteria at multiplicity of infection (MOI) 20∶1. In all infection experiments, bacteria were centrifuged for 3 min, 3000 rpm at 25°C to synchronize the adhesion of bacteria to cells. Where indicated, TNF-α was added at a final concentration of 5 ng/ml. MG-132 was added either 1 h prior to or 4 h after bacterial challenge at a final concentration of 10 µM and maintained during the whole period of infection. SP600125, SB203580 or PD98059 were added 30 min before infection and maintained during the period of infection. After infection, samples were washed extensively before processing. As apoptotic level was more pronounced after 9h of infection [22] with the ST-11 isolates, this time point was therefore undertaken for most experiments, unless otherwise indicated.

### Nuclear extraction and electrophoresis mobility gel shift assay (EMSA)

Nuclear extracts were prepared from uninfected or infected Hec-1B (at different time points), as described previously [73] and bandshift assay was performed by combining 5 µg of nuclear extract with a ^32^P-labeled oligonucleotide probe corresponding to the NF-κB consensus binding site (Santa Cruz Biotechnology, France) and then run on 5% (wt/vol) polyacrylamide gel in Tris-borate-EDTA buffer [74]. Native gel was dried under vacuum, and scanned in a Molecular Imager Faros FX plus (Bio-Rad, France).

### Luciferase reporter assay

Cells co-transfected with (Igκ)_3_conaluc and pCMVβ plasmids were harvested in 200 µl of lysis buffer (50 mM HEPES (pH 7.6), 150 mM NaCl, 1 mM DTT, 1 mM EDTA, 0.5% NP-40, 10% glycerol, and protease inhibitors cocktail) and were incubated for 20 min at 4°C. The supernatants were collected from cell lysates after centrifugation at 12 000 x *g* for 15 min. The luciferase activity was monitored with 60* µ*l of the cell lysate and 20* µ*l of luciferase assay buffer (85 mM DTT, 0.85 mM K_2_HPO_4_ [pH 7.8], 50 mM ATP and D-luciferin substrate). Luciferase activity was measured using a MicroLumat Plus luminometer (EGG BERTHOLD Technologies, Toiry, France). In parallel, samples were assayed for β-galactosidase activity to normalize for transfection efficiency using the *ortho-*nitrophenyl β-D-galactopyranoside (ONPG)-based assay as described previously [75]. The results are reported as fold induction of relative luciferase units (RLU) over the control cells after normalizing for β-galactosidase activity and protein concentration.

### Quantitative measurement of apoptosis and caspase-3 activity assay

Cells were carefully harvested using cold PBS/0.02% EDTA and washed in PBS. Double staining with FITC-Annexin V (specific for apoptotic cells) and Propidium iodide (PI, specific for necrotic cells) was carried out using the FITC-Annexin V kit (Sigma-Aldrich) following the manufacturer's recommendations. Samples were then analysed by flow cytometry. For some experiments, cells infected in 96-well plates in presence of anti-human TLR4 neutralizing mAb (clone HTA125, Abcam) or irrelevant isotype matched IgG control were washed and stained for apoptotic cells using the colorimetric detection and measurement APOPercentage apoptosis assay kit following manufacturer instructions (Biocolor Ltd, N. Ireland). Levels of caspase-3 activity were determined using the colorimetric caspase-3 assay kit (Sigma, France) as described previously [22].

### TNFR1 surface staining and flow cytometry

Cells were labelled with anti-TNFR1, anti-TLR4 mAbs or irrelevant isotype-matched IgG controls as described elsewhere [22]. Stained samples were subjected to Fluorescence Activated Cell Sorting (FACS) analysis using a FACSCalibur flow cytometer (BD Biosciences, France). Fluorescence was recorded from a total of 10,000 events per sample. The acquired fluorescence data were subsequently analysed using WinMDI 2.8 software.

### Fluorescence microscopy

Cells were allowed to adhere for 24 h on coverslips in 24-well plates. After infection with the previously generated DsRed-expressing meningococcal strains (Supporting Table S2 and [22]), coverslips were fixed in 4% paraformaldehyde in PBS and blocked with 1% normal goat serum in PBS. Anti-TNFR1 mAb was used at 1∶50 and FITC-conjugated goat anti-mouse IgG was used at a dilution of 1∶1000. Coverslips were mounted on microscope slides in DAPI-incorporated ProLong Gold antifade reagent (Invitrogen) to both minimize photobleaching and stain nuclei. Slides were then examined by digital confocal microscopy using Zeiss Axio Imager. D1 fluorescent microscope coupled to AxioCam MRm vers.3 (Carl Zeiss, Germany). Digital images were acquired using appropriate filters and combined using the Axiovision Rel. 4.6 software (Carl Zeiss).

### TNF-*α* and soluble TNFR1 (sTNFR1) specific enzyme linked Immuno-sorbant assay (ELISA)

Quantitation of TNF-*α* or sTNFR1 in the culture supernatants was performed by specific ELISA as described elsewhere [22].

### Immunoblotting

Cell lysates fractionated by SDS-PAGE, were transferred to polyvinylidene difluoride (PVDF) membrane which was then probed with primary antibody. The immunoreactive band was visualized using appropriate secondary HRP-conjugated secondary IgG antibody and ECL detection reagents (Amersham Pharmacia Biotech, France). The membranes were visualized using *ChemiDoc XRS* imager and QuantityOne 4.6 software (Bio-Rad).

### Statistical analysis

The *t* test was used to compare two groups, and *P* values<0.05 were considered statistically significant.

## Supporting Information

Figure S1 **Analysis of total and surface level of TLR4 in Hec-1B cells infected with LNP19995 (ST-11) or LNP21019 (carriage) isolates.** Hec-1B cells were infected for the indicated time periods or left uninfected. After incubation cells were harvested, and either lysed and resolved in SDS-PAGE for TLR4 immunoblot analysis in parallel to actin (loading control) (*upper panel*) or stained for analysis of TLR4 surface expression by FACS (*lower panel*). The mean fluorescence intensity (MFI) for each condition is indicated as insert in the histogram plots corresponding to each time point. Immunoblots and FACS data are representative of two independent experiments which yielded similar results.
(TIF)

Figure S2 **Kinetics of NF-κB DNA-binding activity in Hec-1B cells infected with LNP19995 (invasive ST-11) or LNP21019 (carriage) isolates as detected by EMSA.** Hec-1B cells were infected with either strain. The NF-κB DNA binding activity was determined at different time points of infection. Arrowheads represent NF-κB-DNA probe complex or free DNA probe as indicated. The relative intensity of DNA/protein complex for each time point is indicated below each well. EMSA is representative for two independent experiments.
(TIF)

Figure S3 **NF-κB and JNK activation by purified LOS.** Hec-1B cells were treated with LOS purified from the invasive ST-11 isolate LNP19995 or the carriage isolate LNP21019. Cells were harvested after each time point and NF-κB DNA-binding (A) and transactivation (B) were analyzed in parallel to JNK activation (C) as described in Materials and Methods
(TIF)

Figure S4 **Analysis of NF-κB expression in Hec-1B cells infected with LNP19995 (ST-11) or LNP21019 (carriage) isolates.** Hec-1B cells were infected for the indicated time periods. After incubation cells were harvested, and total cell lysates were resolved in SDS-PAGE for NF-κB p65 (*upper panel*), NF-κB p50 (*middle panel*) subunits immunoblot analysis using polyclonal antibodies specific for each subunit. β-Actin expression (*lower panel*) was used as loading control. Immunoblot is representative of three independent experiments which yielded similar results.
(TIF)

Figure S5 **Alteration of NF-κB activation in infected Hec-1B cells impaired secretion of TNF-α.** Hec-1B cells were either pretreated with DMSO (open bars), MG-132 (grey bars) or transfected with pcDNA3 empty vector control (hatched bars) or the vector expressing the dnMyD88 (black bars). Cells were then infected for 9 h and supernatants were collected, cleared from bacteria and assayed for TNF-α using specific ELISA. Values are means ± SD of three independent experiments each performed in duplicates.
(TIF)

Figure S6 **Effect of TACE/ADAM17 inhibition on TNFR1 surface expression.** Hec-1B cells were pre-treated with DMSO or TAPI-1 and then infected in presence or either agent. After 9h of infection, cells were fixed with 4% PFA, permeabilized and stained with anti-TNFR1 mAb and anti-mouse FITC IgG. Nuclei were stained with DAPI. Fluorescence was analyzed using immunofluorescence microscopy. The right panels show enlarged regions of interest from the left merge panels. Note that the loss of TACE activity led to increased expression of TNFR1 at the surface of cells infected with the carriage isolate LNP21019 compared to DMSO-treated cells. White arrowheads indicate cell surface localisation of TNFR1. Scale bar (10 µm) is shown. Data are representative of three independent experiments.
(TIF)

Table S1 ***si*** **RNA oligonucleiotides used in this study.**
(PDF)

Table S2 **Characteristics of invasive ST-11 and carriage isolates of *N. meningitidis* used in this study.**
(PDF)
