# Supplementary figures and images for: Late Repression of NF-κB Activity by Invasive but Not Non-Invasive Meningococcal Isolates Is Required to Display Apoptosis of Epithelial Cells
Source: PLoS Pathog. 2011 Dec 1;7(12):e1002403. doi: 10.1371/journal.ppat.1002403 (PMC3228807; doi:10.1371/journal.ppat.1002403)

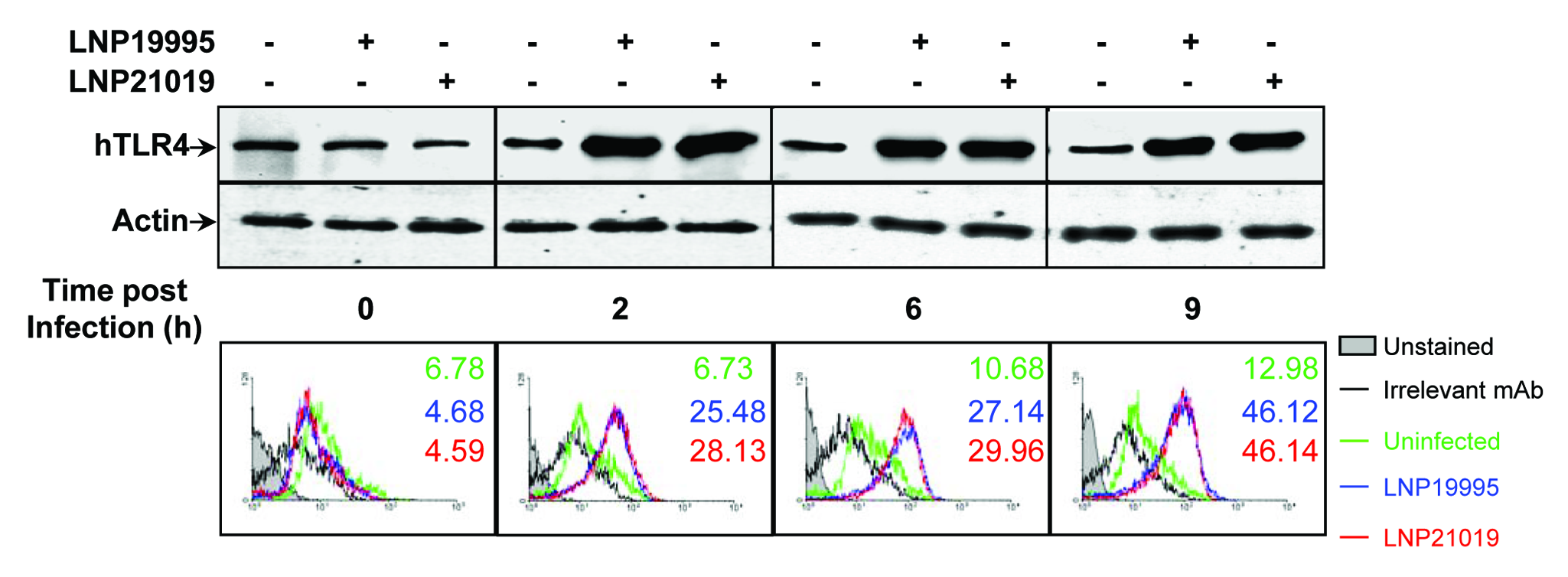

Supplement: Figure S1 — Analysis of total and surface level of TLR4 in Hec-1B cells infected with LNP19995 (ST-11) or LNP21019 (carriage) isolates. Hec-1B cells were infected for the indicated time periods or left uninfected. After incubation cells were harvested, and either lysed and resolved in SDS-PAGE for TLR4 immunoblot analysis in parallel to actin (loading control) (upper panel) or stained for analysis of TLR4 surface expression by FACS (lower panel). The mean fluorescence intensity (MFI) for each condition is indicated as insert in the histogram plots corresponding to each time point. Immunoblots and FACS data are representative of two independent experiments which yielded similar results. (TIF) [file ppat.1002403.s001.tif]

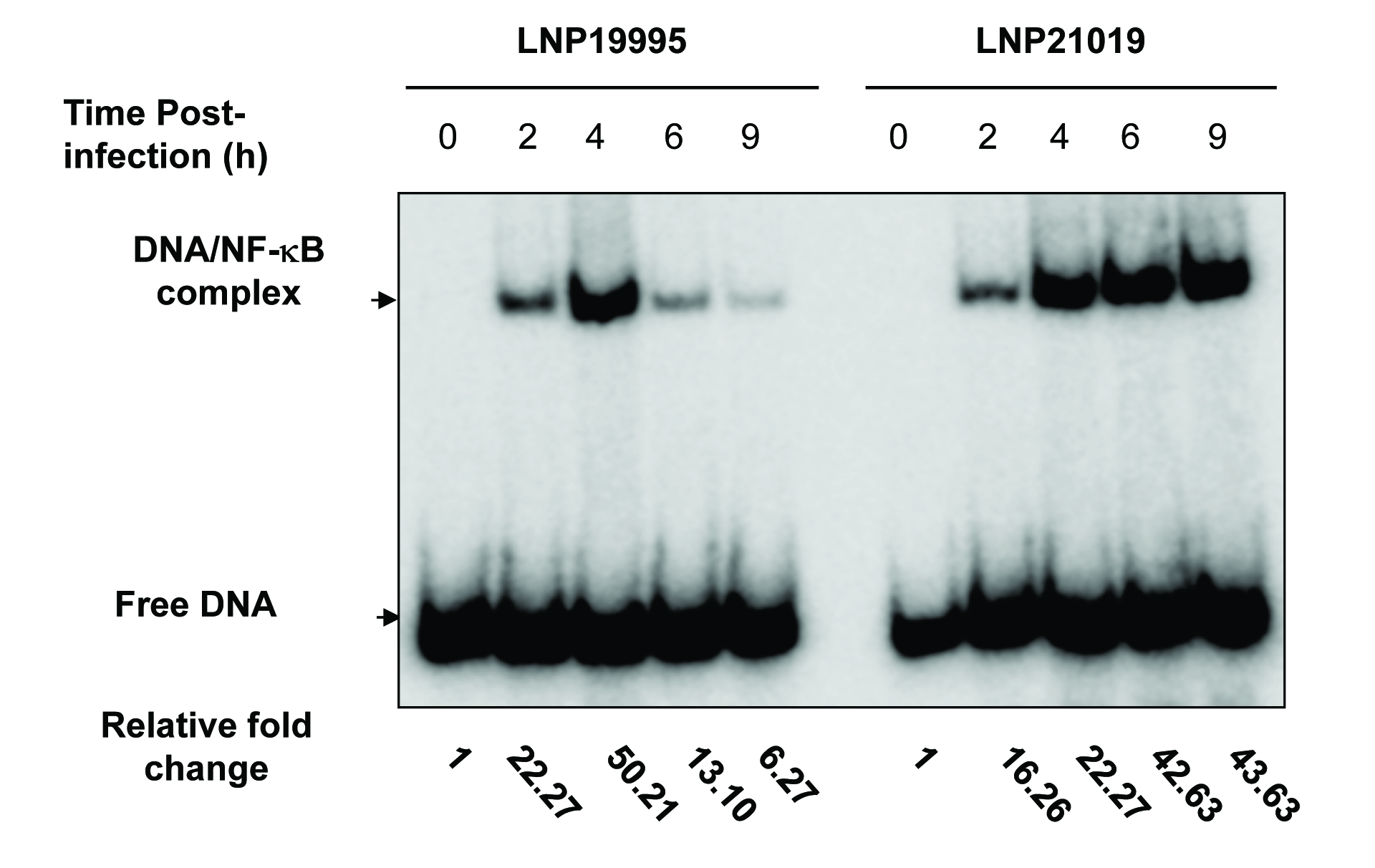

Supplement: Figure S2 — Kinetics of NF-κB DNA-binding activity in Hec-1B cells infected with LNP19995 (invasive ST-11) or LNP21019 (carriage) isolates as detected by EMSA. Hec-1B cells were infected with either strain. The NF-κB DNA binding activity was determined at different time points of infection. Arrowheads represent NF-κB-DNA probe complex or free DNA probe as indicated. The relative intensity of DNA/protein complex for each time point is indicated below each well. EMSA is representative for two independent experiments. (TIF) [file ppat.1002403.s002.tif]

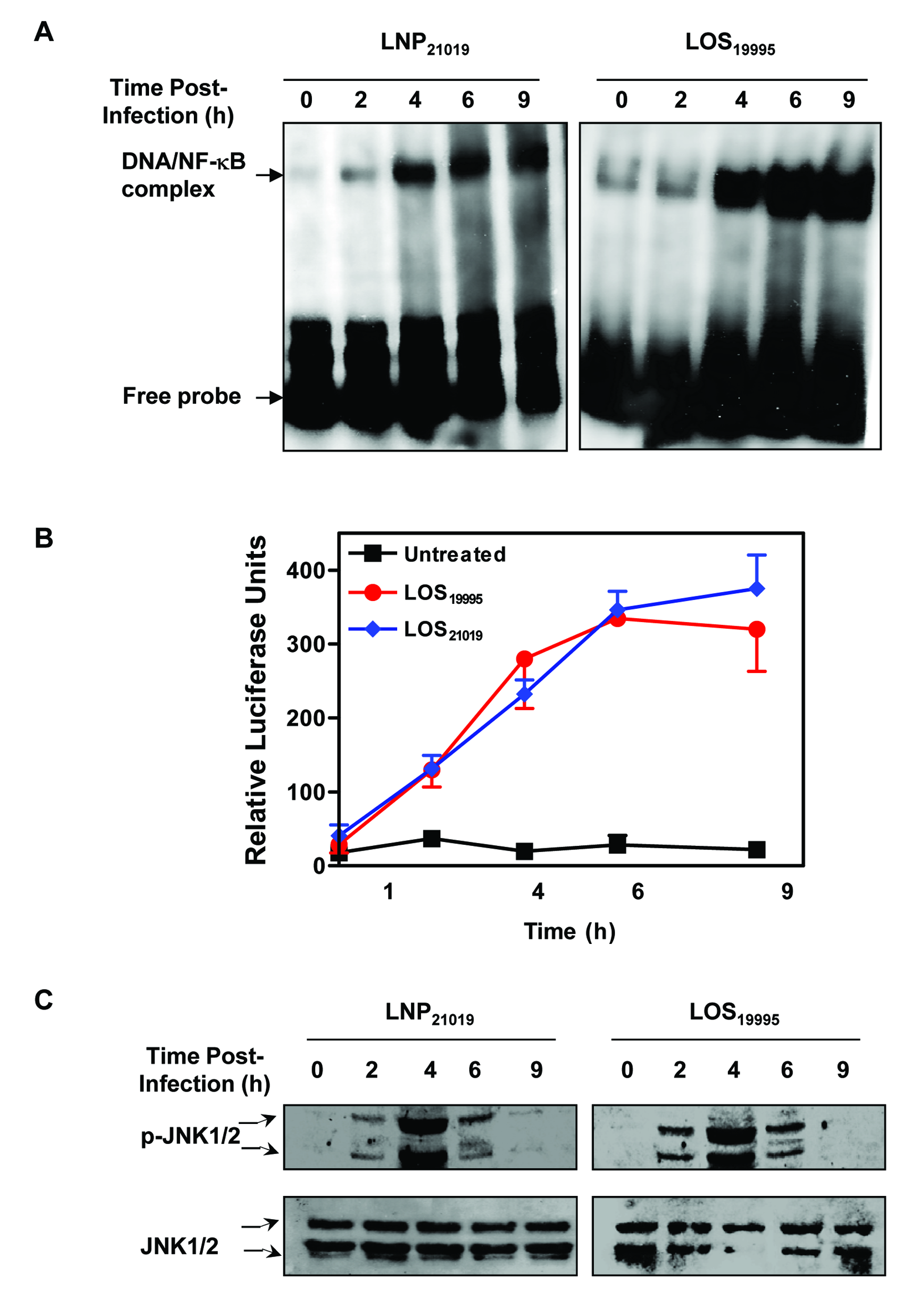

Supplement: Figure S3 — NF-κB and JNK activation by purified LOS. Hec-1B cells were treated with LOS purified from the invasive ST-11 isolate LNP19995 or the carriage isolate LNP21019. Cells were harvested after each time point and NF-κB DNA-binding (A) and transactivation (B) were analyzed in parallel to JNK activation (C) as described in Materials and Methods (TIF) [file ppat.1002403.s003.tif]

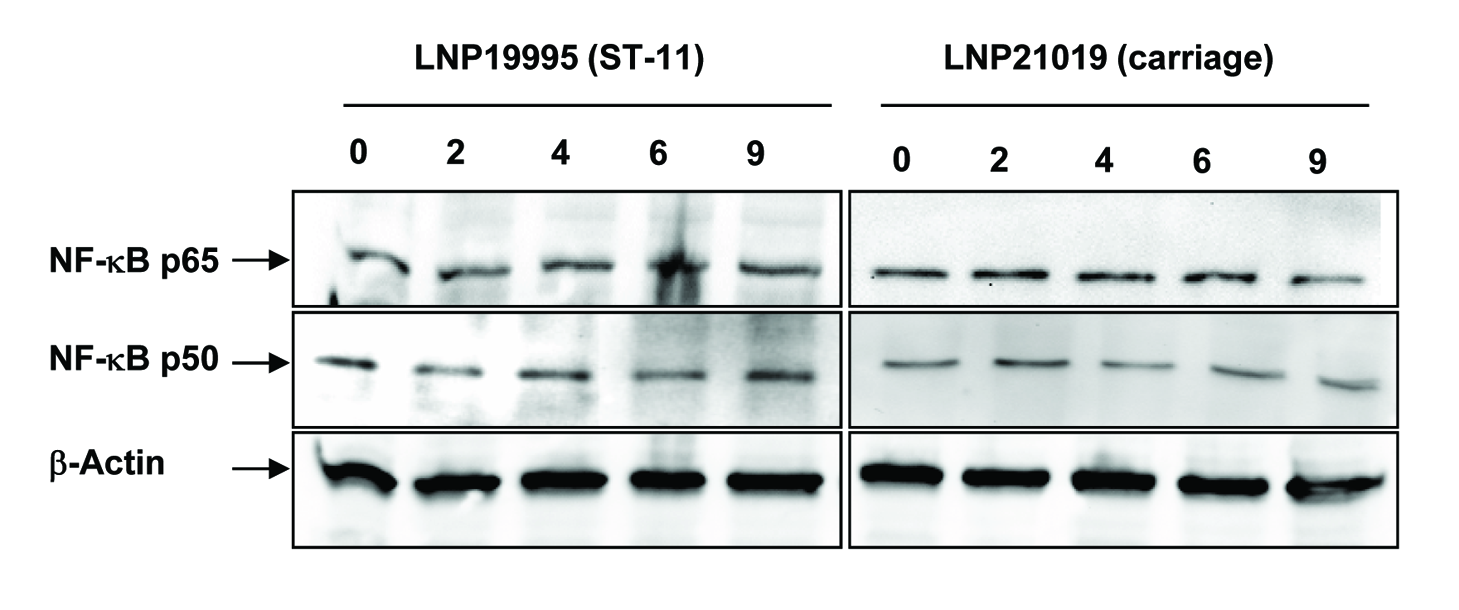

Supplement: Figure S4 — Analysis of NF-κB expression in Hec-1B cells infected with LNP19995 (ST-11) or LNP21019 (carriage) isolates. Hec-1B cells were infected for the indicated time periods. After incubation cells were harvested, and total cell lysates were resolved in SDS-PAGE for NF-κB p65 (upper panel), NF-κB p50 (middle panel) subunits immunoblot analysis using polyclonal antibodies specific for each subunit. β-Actin expression (lower panel) was used as loading control. Immunoblot is representative of three independent experiments which yielded similar results. (TIF) [file ppat.1002403.s004.tif]

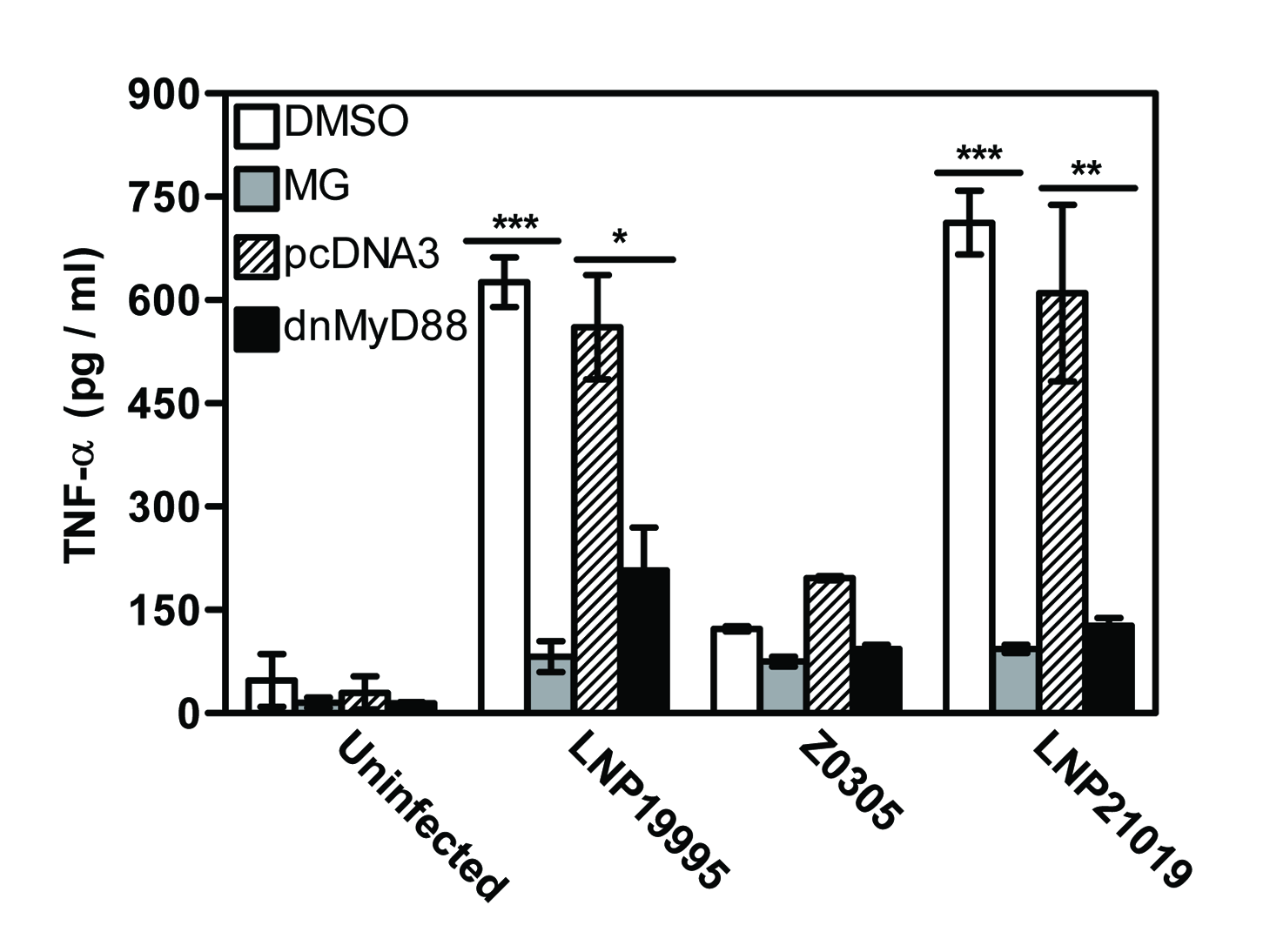

Supplement: Figure S5 — Alteration of NF-κB activation in infected Hec-1B cells impaired secretion of TNF-α. Hec-1B cells were either pretreated with DMSO (open bars), MG-132 (grey bars) or transfected with pcDNA3 empty vector control (hatched bars) or the vector expressing the dnMyD88 (black bars). Cells were then infected for 9 h and supernatants were collected, cleared from bacteria and assayed for TNF-α using specific ELISA. Values are means ± SD of three independent experiments each performed in duplicates. (TIF) [file ppat.1002403.s005.tif]

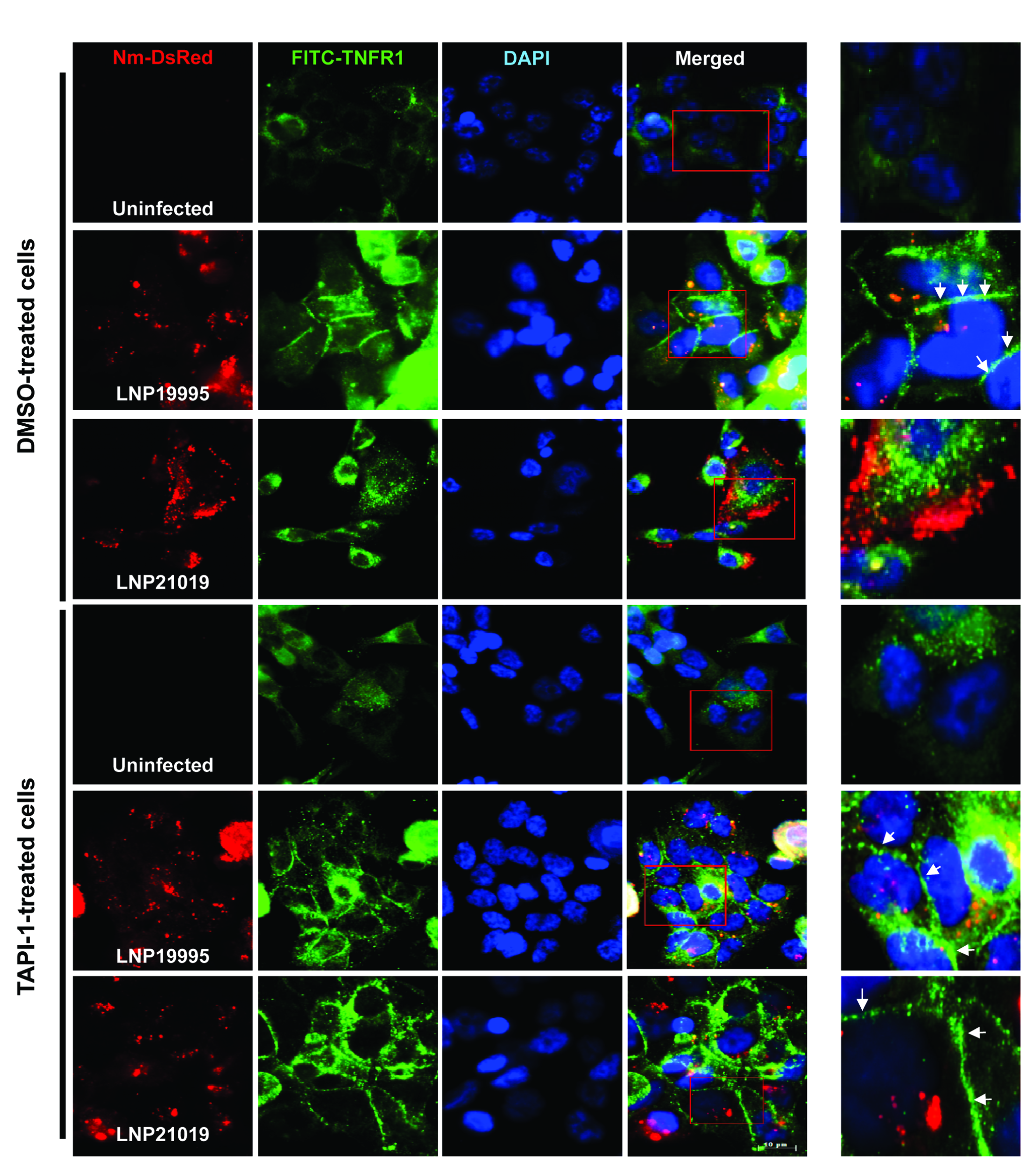

Supplement: Figure S6 — Effect of TACE/ADAM17 inhibition on TNFR1 surface expression. Hec-1B cells were pre-treated with DMSO or TAPI-1 and then infected in presence or either agent. After 9h of infection, cells were fixed with 4% PFA, permeabilized and stained with anti-TNFR1 mAb and anti-mouse FITC IgG. Nuclei were stained with DAPI. Fluorescence was analyzed using immunofluorescence microscopy. The right panels show enlarged regions of interest from the left merge panels. Note that the loss of TACE activity led to increased expression of TNFR1 at the surface of cells infected with the carriage isolate LNP21019 compared to DMSO-treated cells. White arrowheads indicate cell surface localisation of TNFR1. Scale bar (10 µm) is shown. Data are representative of three independent experiments. (TIF) [file ppat.1002403.s006.tif]
