# Supplementary material for: Late Repression of NF-κB Activity by Invasive but Not Non-Invasive Meningococcal Isolates Is Required to Display Apoptosis of Epithelial Cells
Source: PLoS Pathog. 2011 Dec 1;7(12):e1002403. doi: 10.1371/journal.ppat.1002403 (PMC3228807; doi:10.1371/journal.ppat.1002403)
Supplement: Table S1 — si RNA oligonucleiotides used in this study. (PDF) [file ppat.1002403.s007.pdf]

Supporting Table S1: *si*RNA oligonucleiotides used in this study

***si*RNA**	**Sequence**	**Sequence origin and position[1]**
*si*TLR4-1	GGUGUGAAAUCCAGACAAUTT	260-280 (accession number AL160272)
*si*TLR4-2	CGAUGAUAUUAUUGACUUATT	992-1010 (accession number AL160272)
*si*CTRL	UUCUCCGAACGUGUCACGUTT	This study

1: All positions are from start codon
