# Supplementary material for: Late Repression of NF-κB Activity by Invasive but Not Non-Invasive Meningococcal Isolates Is Required to Display Apoptosis of Epithelial Cells
Source: PLoS Pathog. 2011 Dec 1;7(12):e1002403. doi: 10.1371/journal.ppat.1002403 (PMC3228807; doi:10.1371/journal.ppat.1002403)
Supplement: Table S2 — Characteristics of invasive ST-11 and carriage isolates of N. meningitidis used in this study. (PDF) [file ppat.1002403.s008.pdf]

Supporting Table S2: Characteristics of invasive ST-11 and carriage isolates of *N. meningitidis* used in this study.

	Isolate	Clonal Complex	Phenotype	Isolation Site
**Invasive**	LNP13143	ST-11	W135: 2a: P1.5, 2	CSF
	LNP17592	ST-11	W135:2a: P1.5, 2	Blood
	LNP19008	ST-11	C: 2a : P1.5, 2	Blood
	LNP19995	ST-11	W135: 2a: P1.5, 2	CSF
	LNP20342	ST-11	B: 2a: P1-5, 2	CSF
	LNP20553	ST-11	C: 2a: P1.5	CSF
	LNP21515	ST-11	C: 2a: P1.5	CSF
	LNP21678	ST-11	C: 2a: P1.5	Blood
	LNP21996	ST-11	B: 2a: P1. 5	Blood
	LNP24198	ST-11	C: 2a: P1.7,1	Blood
**Carriage**	LNP1026	ST-162	B: NT: P1.4	Nasopharynx
	LNP1046	ST-254	C: NT: P1.4, 7	Nasopharynx
	LNP1288	ST-32	B: 14: P17. 16	Nasopharynx
	LNP1934	ST-32	B: 14: P17. 16	Nasopharynx
	LNP3503	ST-32	B: 14: P1. 7, 16	Nasopharynx
	LNP10820	ST-5	A: 4: P1.9	Expectoration
	LNP16239	NA(1) (ST-1117)	B: NT(2): NST(3)	Expectoration
	LNP18166	ST-22	W135: NT: NST	Expectoration
	LNP20642	ST-334	C: NT: NST	Expectoration
	LNP21019	ST-35	B: NT:P1.14	Expectoration

**Mutant strains**	**Characteristics**
Z0305	Isogenic mutant of the invasive isolate LNP19995, deficient in LOS production (*lpxA::aph3'*), Kan^R [22]
NM0401	Isogenic mutant of the invasive isolate LNP19995, deficient in PorB expression (*porB::erm*), Ery^R [22]
NM0701	Isogenic mutant of the invasive isolate LNP19995, deficient in LOS and PorB expression (*lpxA::aph3'*; *porB::erm*), Kan^R Ery^R [22]
AD1001	Isogenic mutant of the carriage isolate LNP21019, deficient in LOS production (*lpxA::aph3'*), Kan^R [22]
LNP19995 Red	Recombinant strain isogenic of LNP19995 expressing the DsRed fluorescent protein [22]
LNP21019 Red	Recombinant strain isogenic of LNP21019 expressing the DsRed fluorescent protein [22]

(1) NA: not assigned to major clonal complex. The ST between parenthesis represents the sequence type. (2) NT: non-typeable. (3) NST: non-subtypeable. Kan^R: Resistance in kanamycin. Ery^R: resistance in erythromycin.
